# Nuclear PRMT5 is a biomarker of sensitivity to tamoxifen in ERα ^+^ breast cancer

**DOI:** 10.15252/emmm.202217248

**Published:** 2023-07-17

**Authors:** Coralie Poulard, Thuy Ha Pham, Youenn Drouet, Julien Jacquemetton, Ausra Surmielova, Loay Kassem, Benoite Mery, Christine Lasset, Jonathan Reboulet, Isabelle Treilleux, Elisabetta Marangoni, Olivier Trédan, Muriel Le Romancer

**Affiliations:** ^1^ Université de Lyon Lyon France; ^2^ Inserm U1052 Centre de Recherche en Cancérologie de Lyon Lyon France; ^3^ CNRS UMR5286 Centre de Recherche en Cancérologie de Lyon Lyon France; ^4^ Département Prévention et Santé Publique Centre Léon Bérard Lyon France; ^5^ Clinical Oncology Department, Faculty of Medicine Cairo University Cairo Egypt; ^6^ Oncology Department Centre Leon Bérard Lyon France; ^7^ CNRS UMR 5558 LBBE Université de Lyon Villeurbanne France; ^8^ Lipics Services Lyon France; ^9^ Pathology Department Centre Leon Bérard Lyon France; ^10^ Translational Research Department Institut Curie Paris France

**Keywords:** arginine methylation, estrogen receptor, PRMT5, resistance, tamoxifen, Biomarkers, Cancer

## Abstract

Endocrine therapies targeting estrogen signaling, such as tamoxifen, have significantly improved management of estrogen receptor alpha (ERα)‐positive breast cancers. However, their efficacy is limited by intrinsic and acquired resistance to treatment, and there is currently no predictive marker of response to these anti‐estrogens to guide treatment decision. Here, using two independent cohorts of breast cancer patients, we identified nuclear PRMT5 expression as an independent predictive marker of sensitivity to tamoxifen. Mechanistically, we discovered that tamoxifen stimulates ERα methylation by PRMT5, a key event for its binding to corepressors such as SMRT and HDAC1, participating in the inhibition of the transcriptional activity of ERα. Although PRMT5 is mainly localized in the cytoplasm of tumor cells, our analyses show that tamoxifen triggers its nuclear translocation in tamoxifen‐sensitive tumors but not in resistant ones. Hence, we unveil a biomarker of sensitivity to tamoxifen in ERα‐positive breast tumors that could be used to enhance the response of breast cancer patients to endocrine therapy, by fostering its nuclear expression.

## Graphical abstract

The analyses of PRMT5 expression in two large cohorts of luminal breast cancer samples and functional studies revealed a key role of PRMT5 expression in the nucleus of tumor cells in premenopausal women treated with tamoxifen.

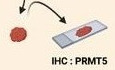

**The paper explained**

**Problem**

Tamoxifen is widely used as anti‐estrogen to treat ERα‐positive breast cancers in premenopausal women. However, 25% of patients relapse due to resistance to treatment, and there is currently no predictive biomarker for tamoxifen sensitivity. A better understanding of the mechanisms underlying resistance is essential to develop innovative strategies to improve clinical outcome for patients with endocrine‐resistant cancers.

**Results**

Our study showed in two independent cohorts of breast tumors that a high nuclear expression of PRMT5 is associated with a prolonged survival of tamoxifen‐treated patients. We also identified nuclear PRMT5 as a key factor in the effects of tamoxifen. Importantly, *in vivo* experiments revealed that tamoxifen triggers PRMT5 translocation to the nucleus of tumor cells where it methylates ERα. This event is a prerequisite for the recruitment of transcriptional corepressors to the promoters of target genes triggering repression of transcription and subsequent decrease in cell proliferation.

**Impact**

These results may have potential clinical application at diagnosis as a high nuclear PRMT5 expression constitutes a predictive biomarker of tamoxifen sensitivity for premenopausal women. Future investigations may help to determine a way to target PRMT5 to the nucleus and thus improve tamoxifen sensitivity.

## Introduction

Breast cancer (BC) is the most common cancer among women worldwide (Bray *et al*, 2018). More than 75% of breast tumors express ERα and belong to the group of luminal BCs. ERα plays a major role in BC tumorigenesis as it regulates cell proliferation and cell survival. Interfering with the ERα pathway using anti‐estrogens (selective estrogen receptor modulators), such as tamoxifen (Tam), or estrogen deprivation (e.g., aromatase inhibitors—AI), increases the survival of ERα‐positive BC patients. Tam remains the standard treatment for premenopausal women with early BC. Despite the high level of sensitivity of luminal tumors to endocrine therapy, treatment efficacy is limited by intrinsic and acquired resistance (Hanker *et al*, 2020). Indeed, 25% of patients relapse during or after the adjuvant endocrine treatment and eventually die from metastases. The main mechanisms underlying intrinsic resistance to Tam are the lack of ERα expression and failure to convert Tam into its active metabolite, whereas acquired resistance has been associated with a plethora of mechanisms. Those include alteration in ERα signaling, crosstalk with growth factors, activation of PI3K/Akt/mTOR pathway, and aberrant expression of cell cycle regulators (Musgrove & Sutherland, 2009). However, none of these mechanisms are routinely assessed to predict Tam sensitivity and guide clinicians towards alternative treatments, highlighting the need for predictive biomarkers (Rasha *et al*, 2021).

The development of many cancers has been attributed to aberrant protein post‐translational modifications (PTMs), resulting from dysregulated gene expression and signaling. Among these PTMs, methylation of arginine residues performed by arginine methyltransferases (PRMTs) is emerging as an important player in cancer (Malbeteau *et al*, 2022). PRMT5, the main type II enzyme, performs monomethylation and symmetrical dimethylation (SDMA). In normal conditions, with its cofactor MEP50, PRMT5 is involved in transcription, ribosome biogenesis, splicing, DNA repair and signal transduction, by methylating histones and nonhistone proteins (Guccione & Richard, 2019).

PRMT5 has oncogenic properties, and its level of expression is high in various cancers. Its overexpression in experimental models increases cancer cell survival, proliferation, migration, and metabolism, and inhibits apoptosis (Poulard *et al*, 2016). PRMT5 has been detected in the nuclear and cytoplasmic compartments and near the cell membrane (Koh *et al*, 2015). Several observations suggest that the subcellular localization of PRMT5 impacts its function. Indeed, cytoplasmic PRMT5 appears to be associated with highly proliferative, less‐differentiated cells, whereas its nuclear expression is correlated with cell cycle arrest and cellular differentiation. In self‐renewing embryonic cells, cytoplasmic PRMT5 plays a crucial role in maintaining the undifferentiated state and relocates to the nucleus upon differentiation (Tee *et al*, 2010). In addition, the cytoplasmic level of PRMT5 is higher in triple‐negative BC (the most aggressive form of BC) (Vinet *et al*, 2019). Our previously published study demonstrated that high levels of nuclear PRMT5 were correlated with increased patient survival with BC (Lattouf *et al*, 2019).

Here, we correlated nuclear PRMT5 expression with sensitivity to Tam in two independent cohorts of ERα^+^ BC cells, and demonstrated that nuclear ERα methylation by PRMT5 is an important step in the transcriptional repression linked with cell growth inhibition induced by Tam.

## Results

### Characteristics of the two BC patient cohorts according to nuclear PRMT5 *H*‐score

**Figure EV1:**
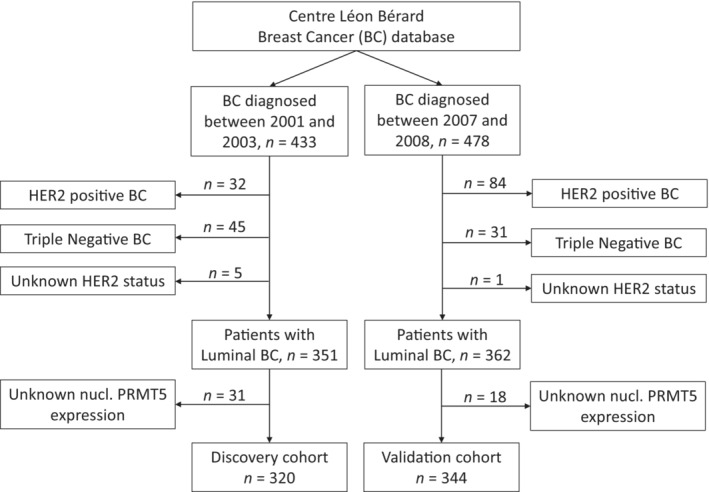
Flowchart describing the Discovery and Validation cohorts

In a previous study, we described that in breast tumor samples, PRMT5 showed a dual expression in the cytoplasm and the nucleus of tumor cells (Lattouf *et al*, 2019). Although it was expressed in the cytoplasm of all tumors, its nuclear expression varied among samples. We showed that high nuclear PRMT5 levels (*H*‐score > 70) were significantly associated with longer relapse‐free survival. Here, two independent cohorts of BC patients were used to analyze the predictive value of nuclear PRMT5 in luminal tumors. The identification of luminal BC was performed according to the flowchart (Fig EV1). The Discovery cohort included 320 patients and the Validation cohort 344, the characteristics of all these patients are presented in Table 1.

**Table 1:** Clinicopathological characteristics of patients at diagnosis for the Discovery and Validation cohorts, according to nuclear PRMT5 *H*‐score.

	Discovery cohort (*n* = 320)				Validation cohort (*n* = 344)				*P*‐value^b^
	Nuclear PRMT5 *H*‐score				Nuclear PRMT5 *H*‐score				
	Low (≤ 70)	High (> 70)	All patients	*P*‐value^a^	Low (≤ 70)	High (> 70)	All patients	*P*‐value^a^	
Age at diagnosis				0.174				**< 0.001**	0.230
Mean (SD)	57.08 (13.16)	59.13 (11.82)	58.54 (12.24)		55.52 (11.87)	61.73 (12.01)	57.40 (12.23)		
Min.–Max.	25–83	33–91	25–91		25–88	30–86	25–88		
Age at diagnosis (cat.)				0.464				**< 0.001**	0.144
≤ 50	32 (34.8%)	64 (28.1%)	96 (30.0%)		92 (38.3%)	20 (19.2%)	112 (32.6%)		
[50–65[	32 (34.8%)	92 (40.4%)	124 (38.8%)		100 (41.7%)	48 (46.2%)	148 (43.0%)		
[65+	28 (30.4%)	72 (31.6%)	100 (31.2%)		48 (20.0%)	36 (34.6%)	84 (24.4%)		
Menopausal status				0.476				**0.005**	0.160
Missing	4	3	7		0	0	0		
Pre	27 (30.7%)	60 (26.7%)	87 (27.8%)		90 (37.5%)	23 (22.1%)	113 (32.8%)		
Post	61 (69.3%)	165 (73.3%)	226 (72.2%)		150 (62.5%)	81 (77.9%)	231 (67.2%)		
BMI				0.894				**0.015**	0.124
Missing	2	10	12		7	3	10		
Mean (SD)	24.48 (4.76)	24.39 (4.94)	24.42 (4.88)		24.58 (4.58)	26.01 (5.52)	25.01 (4.92)		
Min.–Max.	17.10–44.14	16.44–47.56	16.44–47.56		15.00–41.00	17.00–41.00	15.00–41.00		
BMI (cat.)				0.809				**0.017**	0.587
Missing	2	10	12		7	3	10		
≤ 18.5	2 (2.2%)	10 (4.6%)	12 (3.9%)		11 (4.7%)	3 (3.0%)	14 (4.2%)		
[18.5–25[	55 (61.1%)	131 (60.1%)	186 (60.4%)		133 (57.1%)	52 (51.5%)	185 (55.4%)		
[25–30[	23 (25.6%)	53 (24.3%)	76 (24.7%)		66 (28.3%)	23 (22.8%)	89 (26.6%)		
[30+	10 (11.1%)	24 (11.0%)	34 (11.0%)		23 (9.9%)	23 (22.8%)	46 (13.8%)		
Progesterone receptor				0.896				0.723	0.719
Negative	12 (13.0%)	31 (13.6%)	43 (13.4%)		31 (12.9%)	12 (11.5%)	43 (12.5%)		
Positive	80 (87.0%)	197 (86.4%)	277 (86.6%)		209 (87.1%)	92 (88.5%)	301 (87.5%)		
SBR grade				**0.048**				**0.011**	0.907
I	18 (19.6%)	53 (23.2%)	71 (22.2%)		62 (25.8%)	17 (16.3%)	79 (23.0%)		
II	43 (46.7%)	128 (56.1%)	171 (53.4%)		117 (48.8%)	69 (66.3%)	186 (54.1%)		
III	31 (33.7%)	47 (20.6%)	78 (24.4%)		61 (25.4%)	18 (17.3%)	79 (23.0%)		
Surgery type				0.074				0.821	**< 0.001**
Mastectomy	33 (35.9%)	59 (25.9%)	92 (28.8%)		103 (42.9%)	46 (44.2%)	149 (43.3%)		
Tumorectomy	59 (64.1%)	169 (74.1%)	228 (71.2%)		137 (57.1%)	58 (55.8%)	195 (56.7%)		
Pathological T				0.131				0.397	**< 0.001**
Missing	1	7	8		0	0	0		
T0	10 (11.0%)	34 (15.4%)	44 (14.1%)		0 (0%)	0 (0%)	0 (0%)		
T1	45 (49.5%)	129 (58.4%)	174 (55.8%)		128 (53.3%)	62 (59.6%)	190 (55.2%)		
T2	26 (28.6%)	41 (18.6%)	67 (21.5%)		62 (25.8%)	20 (19.2%)	82 (23.8%)		
T3‐T4	10 (11.0%)	17 (7.7%)	27 (8.7%)		50 (20.8%)	22 (21.2%)	72 (20.9%)		
Pathological N				0.920				0.489	**< 0.001**
N0	39 (42.4%)	92 (40.4%)	131 (40.9%)		115 (47.9%)	57 (54.8%)	172 (50.0%)		
N1	41 (44.6%)	103 (45.2%)	144 (45.0%)		61 (25.4%)	22 (21.2%)	83 (24.1%)		
N2‐N3	12 (13.0%)	33 (14.5%)	45 (14.1%)		64 (26.7%)	25 (24.0%)	89 (25.9%)		
Metastasis at diagnosis				‐				‐	‐
M0	92 (100.0%)	228 (100.0%)	320 (100.0%)		240 (100.0%)	104 (100.0%)	344 (100.0%)		
Pathological stage				0.982				0.868	**< 0.001**
I	31 (33.7%)	75 (32.9%)	106 (33.1%)		78 (32.5%)	36 (34.6%)	114 (33.1%)		
II	44 (47.8%)	109 (47.8%)	153 (47.8%)		71 (29.6%)	28 (26.9%)	99 (28.8%)		
III	17 (18.5%)	44 (19.3%)	61 (19.1%)		91 (37.9%)	40 (38.5%)	131 (38.1%)		
Lymphovascular invasion				0.065				0.810	0.092
No	47 (51.1%)	142 (62.3%)	189 (59.1%)		156 (65.0%)	69 (66.3%)	225 (65.4%)		
Yes	45 (48.9%)	86 (37.7%)	131 (40.9%)		84 (35.0%)	35 (33.7%)	119 (34.6%)		
Chemotherapy				0.157				**0.005**	0.479
No	42 (45.7%)	124 (54.4%)	166 (51.9%)		106 (44.2%)	63 (60.6%)	169 (49.1%)		
Yes	50 (54.3%)	104 (45.6%)	154 (48.1%)		134 (55.8%)	41 (39.4%)	175 (50.9%)		
Radiotherapy				0.143				0.346	**0.007**
No	1 (1.1%)	10 (4.4%)	11 (3.4%)		18 (7.5%)	11 (10.6%)	29 (8.4%)		
Yes	91 (98.9%)	218 (95.6%)	309 (96.6%)		222 (92.5%)	93 (89.4%)	315 (91.6%)		
Hormonotherapy				0.089				**< 0.001**	**< 0.001**
Missing	0	5	5		4	3	7		
Tamoxifene exclusive	48 (52.2%)	93 (41.7%)	141 (44.8%)		89 (37.7%)	16 (15.8%)	105 (31.2%)		
Aromatase inhibitor (± preceeded with Tam)	44 (47.8%)	130 (58.3%)	174 (55.2%)		147 (62.3%)	85 (84.2%)	232 (68.8%)		

Bold values denote statistical significance at the *P* < 0.05 level.

^a^ Test comparing low/high PRMT5 distributions in Discovery and Validation cohorts separately.

^b^ Test comparing Discovery and Validation cohorts.

**Figure 1:**
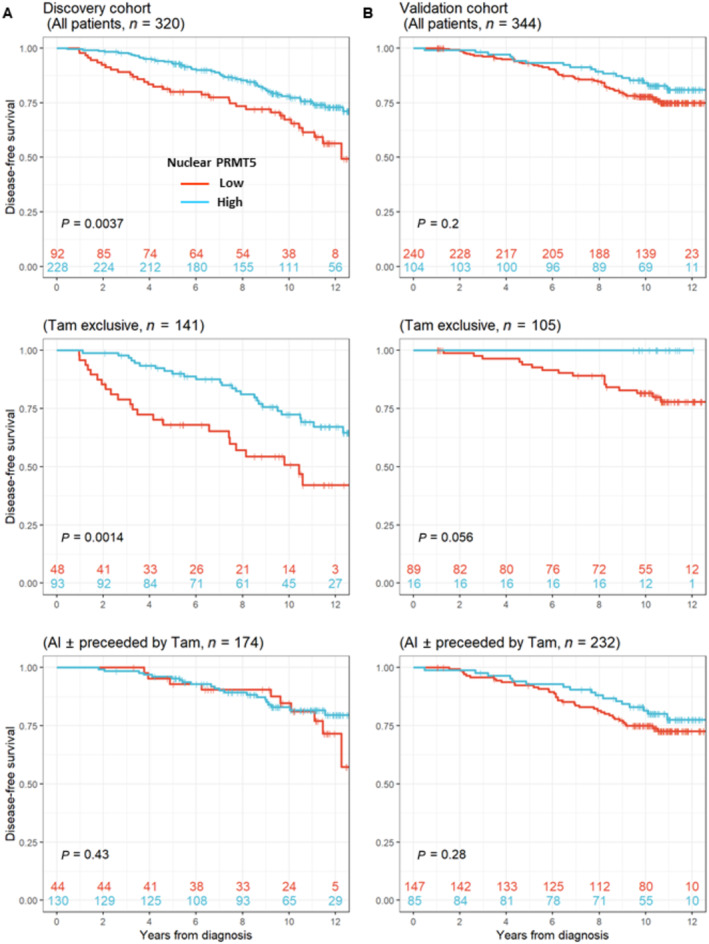
Nuclear PRMT5 is associated with patient survival and interacts with ERα In the Discovery cohort, Kaplan–Meier estimates of disease‐free survival (DFS) in years (y) in patients with low (red) versus high (blue) nuclear PRMT5 expression (upper panel) and in two groups of patients according to their treatment. Patients treated with tamoxifen (Tam, middle panel) and with aromatase inhibitors (AIs, lower panel). *P*‐values are calculated with the logrank test.The same analyses were performed in the Validation cohort. Source data are available online for this figure.

Comparing both cohorts, although overall clinical and pathological characteristics at diagnosis were similar, BCs were diagnosed at a more advanced stage in the Validation cohort than in the Discovery cohort (stage III 38.1 vs. 19.1%). Of note, patients from the Validation cohort were more often treated with Aromatase inhibitor (68.8 vs. 55.2% in the Discovery cohort).

Using an *H*‐score cutoff for nuclear PRMT5 of 70, as previously published (Lattouf *et al*, 2019), we found that in the Discovery cohort, high nuclear PRMT5 expression was statistically associated with low‐grade tumors (*P* = 0.048). In the Validation cohort, high nuclear PRMT5 expression was also associated with low‐grade tumors (*P* = 0.011), as well as with older age (*P* < 0.001) and higher body‐mass index or BMI (*P* = 0.015) at diagnosis.

### High expression of nuclear PRMT5 is associated with increased survival for patients treated with tamoxifen

In the Discovery cohort, we observed that high nuclear PRMT5 expression was statistically associated with prolonged disease‐free survival (Fig 1A, upper panel). Further analysis confirmed this association for patients treated with adjuvant Tam (Tam exclusive group, logrank test: *P* = 0.0014) but not for patients treated with AI (*P* = 0.43) (Fig 1A, middle and lower panels). In the Validation cohort, using the same *H*‐Score, there was no significant association between nuclear PRMT5 and survival for the whole cohort (Fig 1B, upper panel). This was also the case for the AI ± Tam treatment group (*P* = 0.28) (Fig 1B, middle and lower panels). However, in the Tam exclusive treatment group, no relapse or death was observed in patients with high nuclear PRMT5 expression (*P* = 0.056).

We thus speculated that high nuclear PRMT5 could be of prognostic value, and this was validated through adjusted analyses using Cox regression modeling (Table 2). Indeed, adjusted hazard ratios (HR) for high PRMT5 expression were remarkably similar in the Discovery cohort (HR = 0.55, *P* = 0.014) and Validation cohort (HR = 0.56, *P* = 0.039), giving a combined estimate of 0.55 (95% CI: 0.38–0.79, *P* = 0.001).

**Table 2:** Cox regression modeling of progression‐free survival in the Discovery and Validation cohorts.

	Discovery cohort (*n* = 320)			Validation cohort (*n* = 344)			Meta‐analysis^a^
	*n* (%)	Unadjusted HR (95% CI)	Adjusted HR (95% CI)	*n* (%)	Unadjusted HR (95% CI)	Adjusted HR (95% CI)	Adjusted HR (95% CI)
Nucl. PRMT5 *H*‐score							
Low (≤ 70)	92 (28.8)			240 (69.8)			
High (> 70)	228 (71.2)	**0.53 (0.34–0.82, *P* = 0.004)**	**0.55 (0.34–0.88, *P* = 0.014)**	104 (30.2)	0.71 (0.41–1.20, *P* = 0.201)	**0.56 (0.32–0.97, *P* = 0.039)**	**0.55 (0.38–0.79, *P* = 0.001)**
Age at diagnosis							
≤ 50	96 (30.0)			112 (32.6)			
[50–65[	124 (38.8)	0.87 (0.50–1.52, *P* = 0.625)	**3.51 (1.19–10.39, *P* = 0.023)**	148 (43.0)	0.94 (0.51–1.71, *P* = 0.836)	1.38 (0.49–3.92, *P* = 0.543)	**2.16 (1.02–4.58, *P* = 0.044)**
[65+	100 (31.2)	1.61 (0.95–2.74, *P* = 0.077)	**7.31 (2.34–22.78, *P* = 0.001)**	84 (24.4)	**2.17 (1.22–3.88, *P* = 0.009)**	**3.71 (1.25–11.01, *P* = 0.018)**	**5.13 (2.34–11.26, *P* < 0.001)**
BMI							
[18.5–25[	186 (60.4)			185 (55.4)			
≤ 18.5	12 (3.9)	1.78 (0.64–4.95, *P* = 0.273)	**3.93 (1.30–11.91, *P* = 0.016)**	14 (4.2)	2.30 (0.90–5.84, *P* = 0.080)	2.06 (0.79–5.42, *P* = 0.141)	**2.72 (1.32–5.64, *P* = 0.007)**
[25–30[	76 (24.7)	1.30 (0.77–2.20, *P* = 0.320)	1.14 (0.65–2.00, *P* = 0.636)	89 (26.6)	0.70 (0.38–1.30, *P* = 0.259)	0.53 (0.29–1.00, *P* = 0.05)	0.82 (0.54–1.24, *P* = 0.34)
[30+	34 (11.0)	1.84 (0.99–3.43, *P* = 0.055)	1.65 (0.86–3.17, *P* = 0.133)	46 (13.8)	1.48 (0.80–2.73, *P* = 0.208)	1.19 (0.63–2.25, *P* = 0.593)	1.39 (0.88–2.2, *P* = 0.15)
Menopausal status							
Post	226 (72.2)			231 (67.2)			
Pre	87 (27.8)	1.20 (0.75–1.92, *P* = 0.440)	**3.68 (1.31–10.34, *P* = 0.013)**	113 (32.8)	0.65 (0.38–1.10, *P* = 0.111)	0.99 (0.36–2.77, *P* = 0.992)	1.9 (0.92–3.94, *P* = 0.083)
Pathological stage							
I	106 (33.1)			114 (33.1)			
II	153 (47.8)	**1.94 (1.07–3.52, *P* = 0.028)**	1.66 (0.87–3.19, *P* = 0.127)	99 (28.8)	**2.16 (1.06–4.43, *P* = 0.035)**	**2.22 (1.05–4.68, *P* = 0.037)**	**1.88 (1.15–3.08, *P* = 0.012)**
III	61 (19.1)	**3.88 (2.07–7.26, *P* < 0.001)**	**2.69 (1.32–5.48, *P* = 0.006)**	131 (38.1)	**3.54 (1.86–6.73, *P* < 0.001)**	**3.79 (1.86–7.71, *P* < 0.001)**	**3.19 (1.93–5.28, *P* < 0.001)**
SBR grade							
1	71 (22.2)			79 (23.0)			
2	171 (53.4)	1.43 (0.73–2.80, *P* = 0.295)	1.71 (0.81–3.62, *P* = 0.163)	186 (54.1)	1.44 (0.73–2.81, *P* = 0.293)	1.34 (0.67–2.70, *P* = 0.412)	1.5 (0.9–2.5, *P* = 0.12)
3	78 (24.4)	**3.42 (1.73–6.75, *P* < 0.001)**	**2.42 (1.10–5.34, *P* = 0.029)**	79 (23.0)	**2.50 (1.23–5.08, *P* = 0.011)**	1.93 (0.89–4.20, *P* = 0.096)	**2.16 (1.24–3.76, *P* = 0.006)**
Lymphovascular Inv.							
No	189 (59.1)			225 (65.4)			
Yes	131 (40.9)	**2.47 (1.59–3.83, *P* < 0.001)**	**2.17 (1.27–3.72, *P* = 0.005)**	119 (34.6)	1.55 (0.98–2.46, *P* = 0.061)	1.11 (0.64–1.91, *P* = 0.711)	**1.56 (1.06–2.28, *P* = 0.023)**

Bold values denote statistical significance at the *P* < 0.05 level.

^a^ Multivariable adjusted Cox models from Discovery and Validation cohorts were combined using a fixed‐effects meta‐analysis model.

In order to assess whether the prognostic value of PRMT5 could vary according to endocrine therapy, menopausal status and stage at diagnosis, stratified Cox models, and interaction tests were performed, and the 10‐year survival probability estimated from these models was displayed. Although no statistically significant interaction was found (PRMT5 × hormonotherapy: *P* = 0.148; PRMT5 × menopausal status: *P* = 0.87; PRMT5 × stage: *P* = 0.99), the stratified Cox models highlighted better disease‐free survival for patients with high PRMT5 expression, at all stages, and particularly for stage III patients treated with Tam (Appendix Fig S1).

These observations led us to hypothesize that nuclear PRMT5 could be involved in Tam treatment efficacy, possibly by regulating ERα transcriptional activity.

### ERα/PRMT5 interaction is regulated differently by the ligands

**Figure 2:**
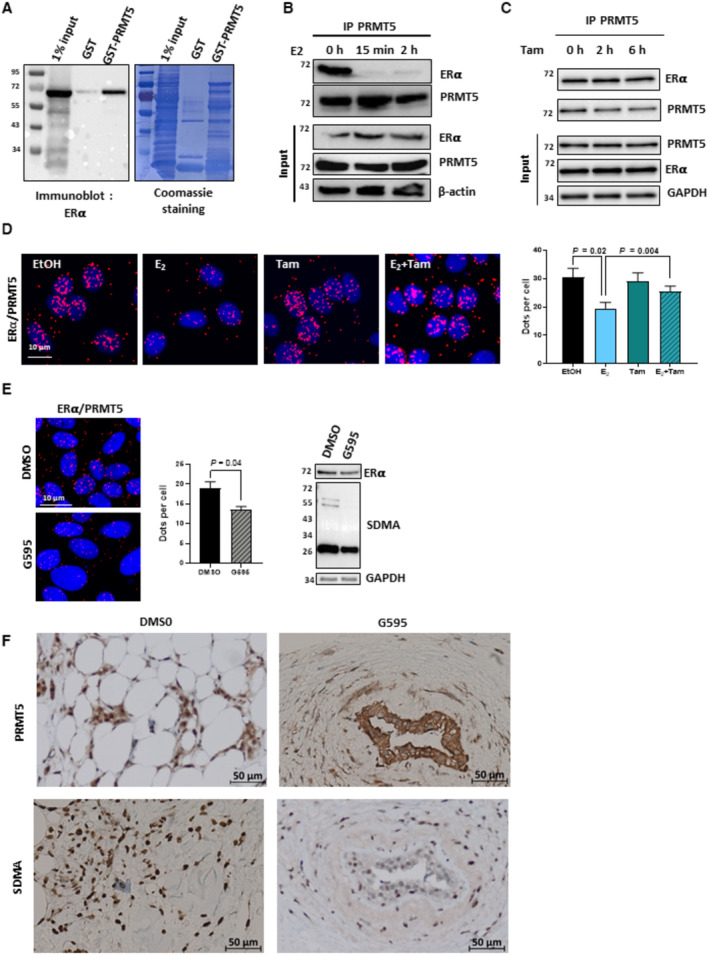
ERα interacts with PRMT5 GST and GST‐PRMT5 fusion proteins were incubated with *in vitro*‐translated ERα, the interaction was then visualized by Western blotting using an anti‐ERα antibody. The corresponding Coomassie‐stained gel is shown in the right panel.ERα/PRMT5 interaction was assessed by co‐immunoprecipitation of MCF7 cell extracts treated or not with E_2_ for the indicated times, using an anti‐PRMT5 antibody. The presence of PRMT5, ERα and β‐actin was evaluated by Western blot analysis.The same experiment was performed for MCF7 cells treated or not with tamoxifen (Tam) for the indicated times. The scale bar is 10 μm.Proximity Ligation Assay (PLA) to detect interactions between ERα and PRMT5 in MCF7 cells treated with E_2_, Tam 1 μM or both for 6 h. After fixation, PLA experiments were performed to evaluate the interactions between ERα/PRMT5 using specific antibodies. The detected dimers are represented by red dots. The nuclei were counterstained with mounting medium containing DAPI (blue) (Obj: X60). Quantification of the number of dots per cell was performed by computer‐assisted analysis as reported in the Materials and Methods section. The mean ± SEM of three independent experiments is shown. *P*‐values were determined using a Student's *t*‐test.The ERα/PRMT5 interaction was studied by PLA as in D with or without the PRMT5 inhibitor G595. The scale bar is 10 μm. Quantification was performed as described above. The mean ± SEM of three independent experiments is shown. *P*‐values were determined using a Student's *t*‐test. Western blotting was performed to assess ERα expression and the global profile of methylation using a pan SDMA antibody.Paraffin‐embedded sections from a fresh tumor were incubated with or without G595, and PRMT5 and SDMA expression were assessed by IHC. Source data are available online for this figure.

To search for ERα interaction with PRMT5, we initially conducted a GST pull‐down experiment, and found that ERα specifically interacts with PRMT5 but not with the GST (Fig 2A). In MCF7 cells, we then conducted immunoprecipitation (IP) assays and found that ERα interacts specifically with endogenous PRMT5, and that E_2_ disrupts this interaction (Fig 2B), while Tam had no effect (Fig 2C).

Next, we investigated in which cellular compartment these interactions occurred by conducting proximity ligation assays (PLA) using different antibody pairs. The images obtained revealed specific red dots predominantly in the nucleus of MCF7 cells, illustrating the interaction between ERα and PRMT5, which decreased upon treatment with E_2_ but remained unchanged with Tam alone and Tam combined to E_2_ (Fig 2D). The signals strongly decreased in MCF7 cells after the downregulation of PRMT5 and ERα, highlighting the specificity of these interactions (Appendix Fig S2). Interestingly, treatment of MCF7 cells with the PRMT5 inhibitor GSK3326595 (herein called G595) significantly decreased ERα/PRMT5 binding. (Fig 2E). To gain further insights, we investigated PRMT5 localization after G595 treatment. Using sections of a paraffin‐embedded fresh breast tumor, we observed that upon G595 treatment, PRMT5 expression in the nucleus significantly decreased (Fig 2F). We also confirmed this result after cell fractionation of MCF7 cells (Appendix Fig S2B).

### ERα is a new substrate for PRMT5

**Figure 3:**
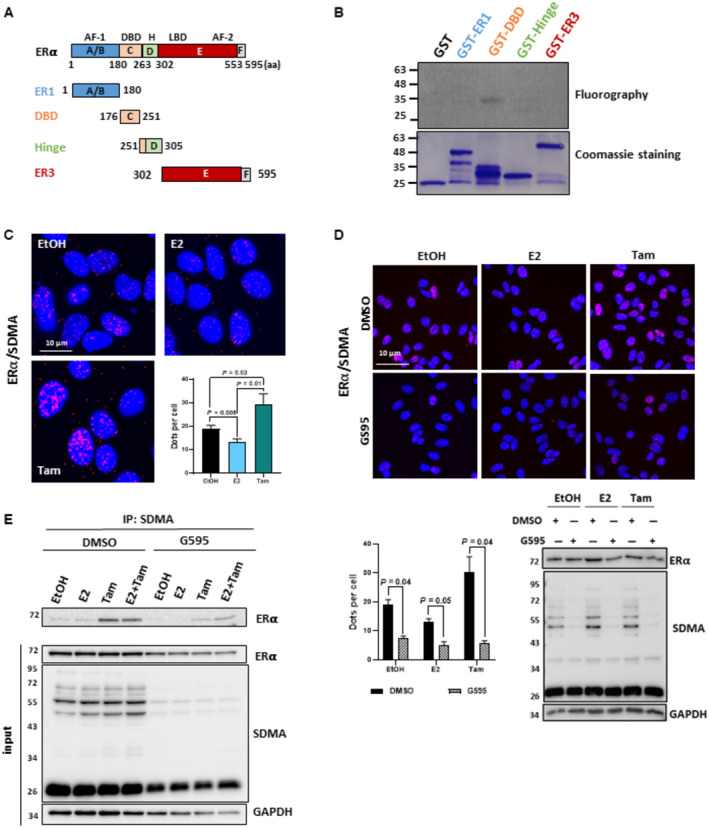
PRMT5 methylates ERα ERα domain: A/B encompassing Activation Function‐1 (AF‐1); C containing the DNA‐binding domain (DBD); D containing nuclear localization signals (NLS); E containing the ligand‐bind domain (LBD) and the Activation‐Function‐2 (AF‐2), and finally the agonist /antagonist regulatory F region.
*In vitro* methylation experiments were performed by incubating the PRMT5/MEP50 complex with [^32P^γ] ATP and GST or the different ERα constructs. The methylated proteins were visualized by auto‐radiography (upper panel). The corresponding Coomassie‐stained gel is shown in the lower panel.MCF7 cells were treated with or without E_2_, Tam or both for 6 h. ERα methylation was determined by PLA using an anti‐ERα and a pan methyl recognizing symmetrical dimethylation (SDMA). The scale bar is 10 μm. The mean ± SEM of three independent experiments is shown. *P*‐values were determined using a Student's *t*‐test.The same experiment was performed with G595 to verify that ERα methylation was PRMT5‐dependent. Red dots reflect ERα methylation. The mean ± SEM of three independent experiments is shown. *P*‐values were determined using a Student's *t*‐test. The sale bar is 40 μm. Western blotting was performed to assess ERα, PRMT5 expression and the global profile of methylation using a pan SDMA antibody.MCF7 cells were treated as in (C) and lysates were immunoprecipitated with a pan SDMA antibody and immunoblotted with an anti‐ERα antibody. ERα, SDMA and GAPDH expression was evaluated in the input. Source data are available online for this figure.

We then performed *in vitro* methylation experiments, to confirm the methylation of ERα by PRMT5. Purified GST‐ERα fragments containing the different structural domains (Fig 3A) were incubated with the active PRMT5 complex. The C region containing the DNA‐binding domain was the only one methylated by PRMT5 (Fig 3B), though the exact arginine residue targeted was not identified. We then applied PLA to assess cellular ERα methylation (Poulard *et al*, 2020) using an antibody raised against ERα and another recognizing symmetrical dimethylation (SDMA); in this case, each red dot represents a methylation event. We observed metERα SDMA in the nucleus of MCF7 cells, and these signals significantly decreased upon E_2_ treatment and increased upon Tam treatment (Fig 3C). The involvement of PRMT5 was confirmed using the PRMT5 inhibitor G595, which impaired SDMA methylation (Fig 3D). Control experiments were performed using siERα or by knocking down PRMT5 or its cofactor MEP50 (Appendix Fig S3A and B). This result was confirmed by IP with an anti‐SDMA antibody revealed by ERα (Fig 3E).

**Figure EV2:**
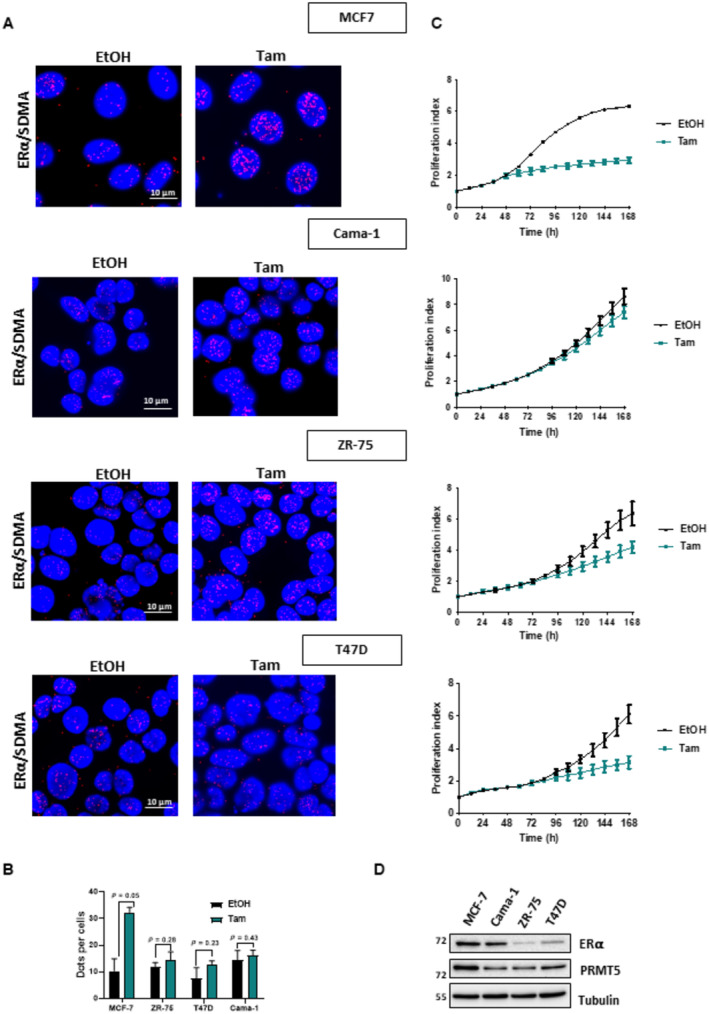
Study of ERα/SDMA in ERα‐positive BC cells treated with tamoxifen MCF7, Cama‐1 and ZR‐75 and T47D cells were treated with Tam (1 μM) or ethanol (EtOH). After fixation, proximity ligation assay (PLA) was performed to evaluate ERα methylation using specific antibodies. The detected dimers are represented by red dots. The nuclei were counterstained with DAPI (blue) (Obj: X60) (left panel).Quantification of the number of dots per cell was performed by computer‐assisted analysis as reported in the Materials and Methods section. The mean ± SEM of one experiment representative of three experiments is shown.The four cell lines were plated onto 96‐well plates and treated with Tam (1 μM) or ethanol (EtOH) and proliferation was measured in real time using the IncuCyte technology. Image acquisition was conducted every hour using the IncuCyte software, which calculates the percentage of cell confluency as a function of time over 7 days. The results are represented as graphs showing the rate of proliferation every 24 h. The mean ± SD of one experiment representative of three experiments is shown.ERα, PRMT5 and Tubulin expression were assessed by Western blotting in the four ERα‐positive BC cells.

When looking at other ERα‐positive BC cells, we saw by PLA that ERα methylation was significantly greater in MCF7 cells, which displayed a stronger inhibition of proliferation upon Tam treatment (Fig EV2A and B). ZR‐75 and T47D cells only partially responded to Tam, and Cama‐1 cells remained largely unaffected (Fig EV2C). We ruled out that this result was not due to the level of ERα expression, as this was similar in MCF7 and Cama‐1 cells (Fig EV2D). Hence, Tam may repress estrogen signaling by impacting PRMT5 methylation of ERα.

**Figure 4:**
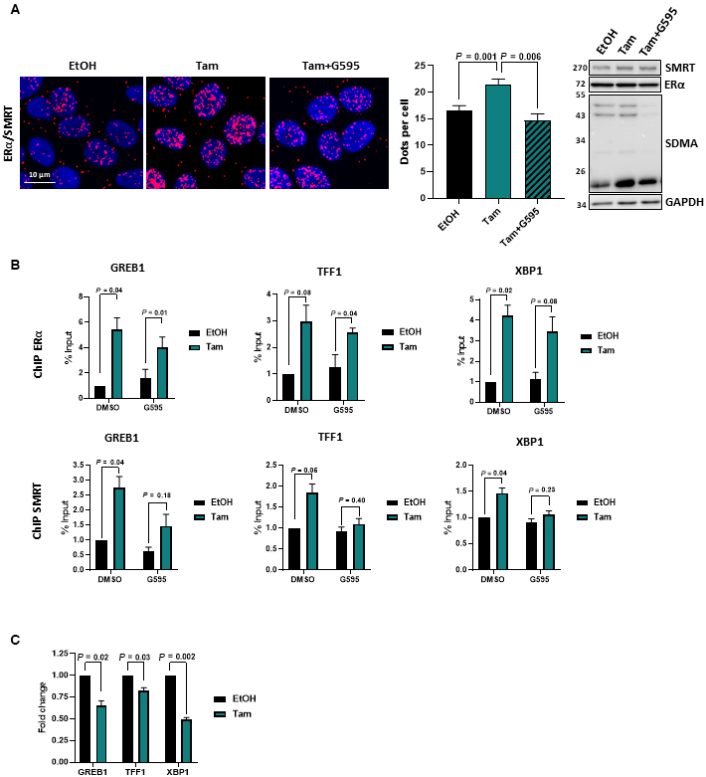
PRMT5 activity is required for the interaction between ERα and the corepressors SMRT MCF7 cells were treated with Tam for 6 h in the presence or absence of G595, the interaction between ERα and SMRT was then determined by PLA using specific antibodies against both proteins. The scale bar is 10 μm. Quantification was performed as in Fig 2 (middle panel). The mean ± SEM of three independent experiments is shown. *P*‐values were determined using a Student's *t*‐test. Western blotting was performed to assess ERα, SMRT and GAPDH expression and the global profile of SDMA.MCF7 cells were treated with Tam for 6 h in the presence or absence of G595, cell extracts were then subjected to ChIP assay using anti‐ERα or anti‐SMRT antibodies. The precipitated DNA fragments were used for qPCR analysis using specific primers for the indicated promoters. The results are expressed relative to the signal obtained from input chromatin. The mean ± SEM of at least three experiments is shown. *P*‐values were determined using a Student's *t*‐test.MCF7 cells were treated with Tam for 6 h and total RNA was prepared and cDNAs were analyzed by RT‐QPCR with specific primers for GREB1. The values were normalized against 28S mRNA and represent the ± SEM of three experiments. Source data are available online for this figure.

### PRMT5 activity is required for the interaction between ERα and transcriptional corepressors

**Figure EV3:**
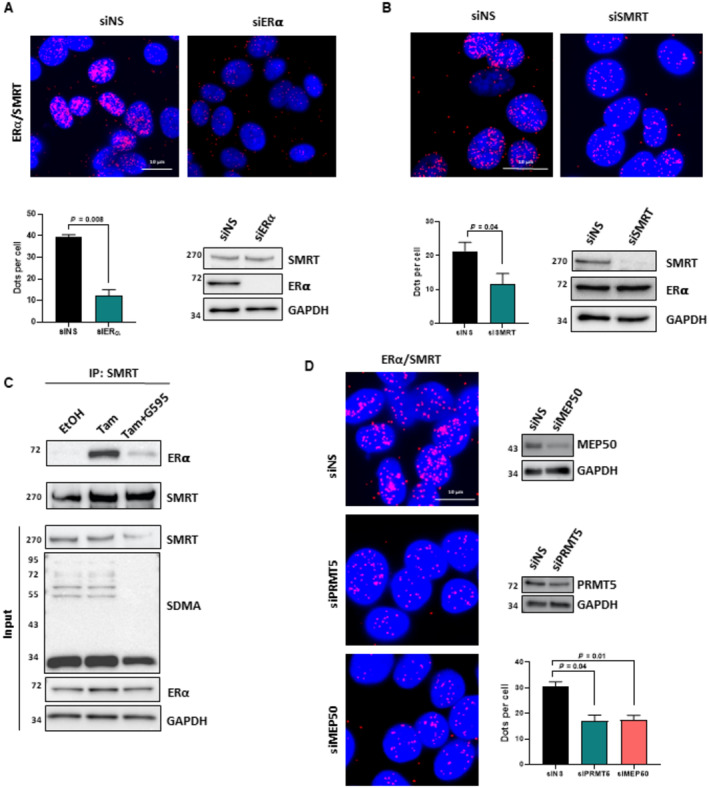
Study of ERα/SMRT interaction in MCF7 cells A, BMCF7 cells were transfected with siNS or siRNAs targeting ERα or siSMRT. After fixation, PLA was performed to evaluate ERα/SMRT interactions using specific antibodies (Obj: X60). Quantification of the number of dots per cell was performed as described above. The efficacy of protein inhibition was verified by Western blotting using the corresponding antibodies.CMCF7 were treated with Tam 1 μM for 6 h in the presence or absence of G595. Cell extracts were then used to immunoprecipitate SMRT. The presence of SMRT and ERα in the inputs were studied by Western blotting together with SDMA and GAPDH.DMCF7 cells were transfected with siNS or siRNAs targeting PRMT5 or MEP50. After siRNA transfection and fixation, PLA was performed to evaluate ERα/SMRT interactions using specific antibodies (Obj: X60). Quantification of the number of dots per cell was performed as described above. The efficacy of protein inhibition was verified by western blotting using the corresponding antibodies. The *P*‐value was determined using a Student *t*‐test.

**Figure EV4:**
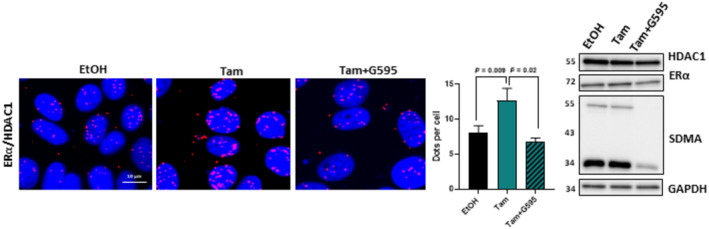
Detection of ERα/HDAC1 interactions in MCF7 cells MCF7 were treated with Tam 1 μM for 6 h in the presence or absence of G595, the cells were then fixed and PLA was performed to study ERα/HDAC1 interactions. Quantification of the number of dots per cell was performed as described above. The efficacy of G595 was verified by Western blotting using the corresponding antibodies.

It is well‐known that estrogen triggers the activation of transcription through the recruitment of a plethora of coactivators, whereas Tam inhibits estrogen by fostering the interaction of ERα with its corepressors, such as SMRT, which recruit histone deacetylases to impede transcription (Shang *et al*, 2000; Liu & Bagchi, 2004; Papachristou *et al*, 2018). In this context, we assessed whether PRMT5 could be involved in the interaction between ERα and such corepressors. By PLA, we showed that upon Tam treatment, ERα/SMRT interactions significantly increased, whereas when cells were treated with G595, this increase was abrogated (Fig 4A). Controls for PLA experiments are shown in Fig EV3A and B. This result was further confirmed by co‐immunoprecipitation (Fig EV3C). In addition, knocking down PRMT5 or MEP50 had the same effect as G595 on the interaction between ERα and SMRT (Fig EV3D). We found the same result for HDAC1, another well‐known ERα corepressor (Liu & Bagchi, 2004) (Fig EV4).

To gain further mechanistic insights into the role of PRMT5 in ERα/SMRT interactions, we then studied the impact of PRMT5 activity on the level of binding of ERα and SMRT at the promoter of three well‐described ERα target genes by chromatin immunoprecipitation (ChIP) (Fig 4B). We found that Tam induced the binding of ERα and SMRT to promoters of *GREB1*, *TFF1*, and *XBP1*. However, in the presence of G595, ERα was still significantly bound to chromatin, whereas SMRT was not recruited to promoters of *GREB1*, *TFF1*, and *XBP1*. We then studied the expression of these three target genes and validated that Tam treatment induced a decrease in the expression of GREB1, TFF1, and XBP1 mRNA (Fig 4C).

**Figure 5:**
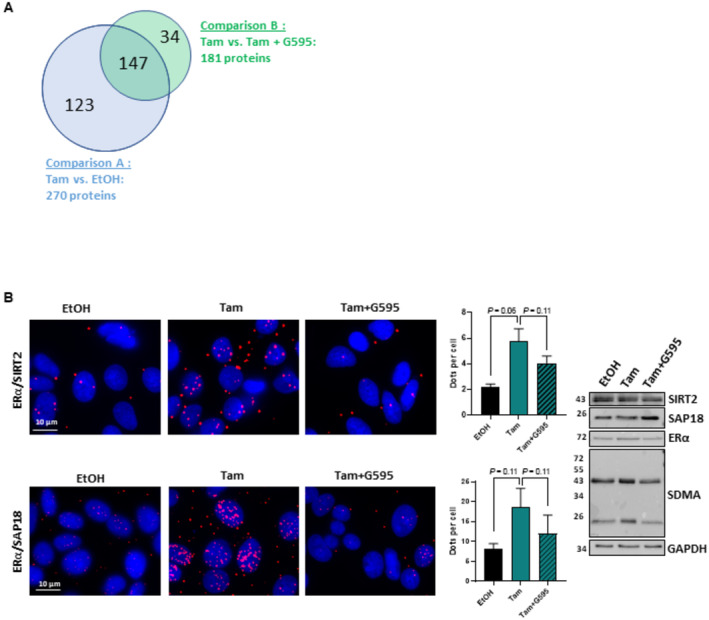
Large scale analysis to search for ERα interactors dependent on Tam and PRMT5 activity Venn diagram of the BIFC screen. Blue Venn diagram represents the genes regulated by Tam in comparison to EtOH. Green Veen diagram represents the genes regulated by G595 + Tam in comparison to Tam alone.MCF7 cells were treated with Tam for 6 h in the presence or in absence of G595, then ERα interaction with SIRT2 and SAP18 was determined by PLA using specific antibodies against both proteins. The scale bar is 10 μM. Quantification was performed as in Fig 2. The mean ± SEM of three independent experiments is shown. *P*‐values were determined using a Student's *t*‐test. Western blotting was performed to assess ERα, SIRT2, SAP18, and GAPDH expression and the global profile of SDMA. Source data are available online for this figure.

**Figure EV5:**
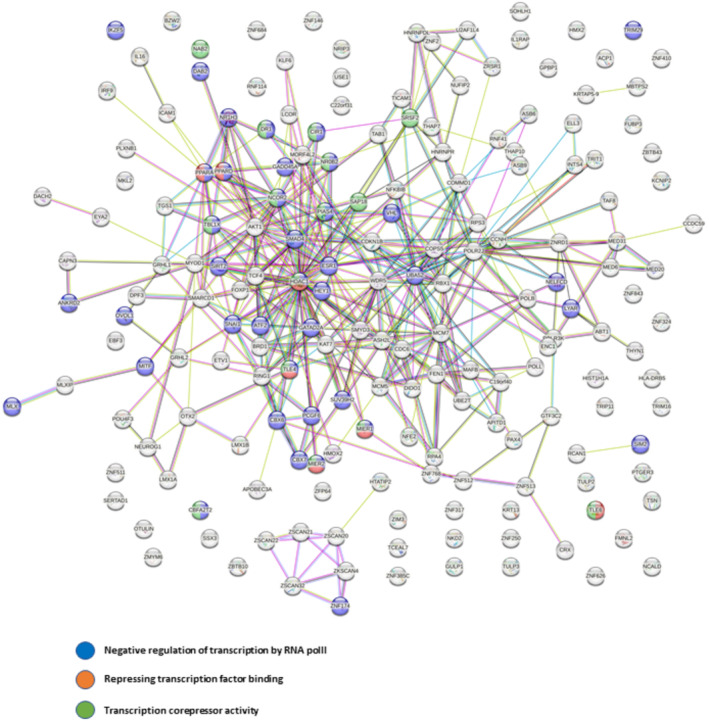
Analysis of protein interactions for ERα partners identified by our screen Proteins whose interactions with ERα were induced by Tam and regulated by PRMT5 activity were analyzed using the STRING database. Proteins involved in the repression of transcription are highlighted in color.

To evaluate whether this mechanism could be generalized, we performed a large‐scale analysis with a screening approach based on bimolecular fluorescence complementation (BIFC) using ERα as a bait. We used this system to assess changes in the network of ERα interactions induced by drugs. Here, we compared the ERα interactome in MCF7 cells treated or not with Tam in the presence or absence of G595. We analyzed ERα interactions induced by Tam treatment (comparison A) and we compared the interactors in the presence of Tam or Tam plus G595 (comparison B) (Fig 5A). We found that among the 1,812 transcription factors tested (Dataset EV1), 270 proteins bound ERα in the presence of Tam and 181 interactions were disrupted by G595. Of note, 54% of interactions induced by Tam were lost with G595, highlighting that PRMT5 activity strongly influences the ERα interactome in the presence of Tam. As expected, a large number of interactors were involved in transcription repression (Fig EV5). We then validated by PLA two ERα interactors; SIRT2 and SAP18 (Fig 5B).

### Tamoxifen enhances ERα methylation *in vivo* by fostering PRMT5 nuclear translocation

**Figure 6:**
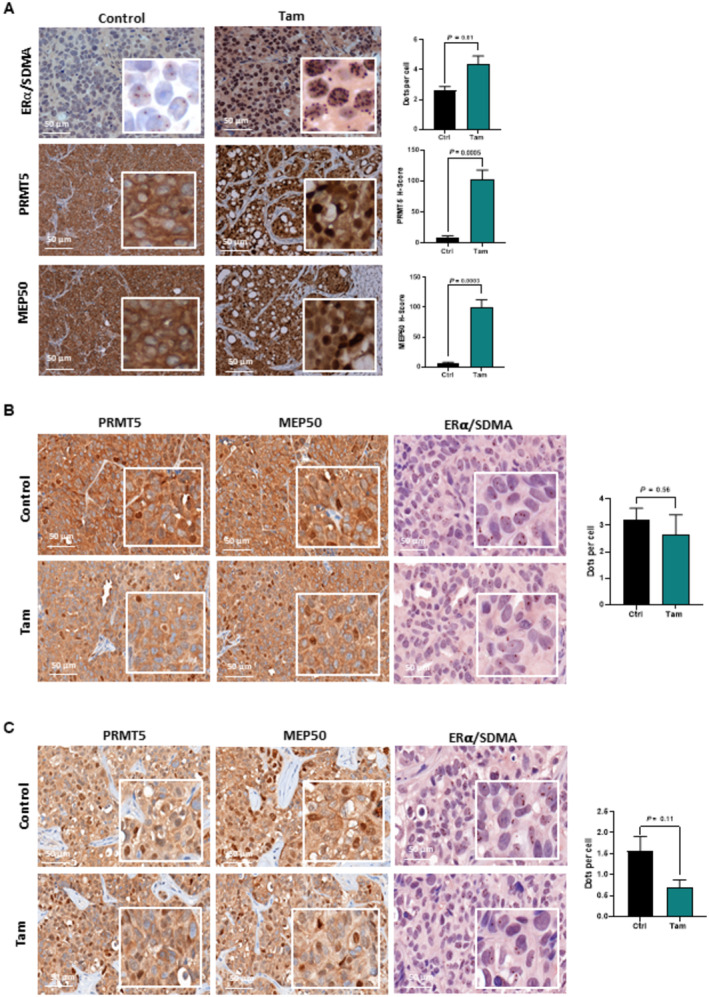
Tamoxifen increases ERα methylation and PRMT5 nuclear localization *in vivo* in Tam‐sensitive tumors ERα methylation SDMA was studied by PLA, and PRMT5 and MEP50 expression were analyzed by IHC on formalin‐fixed MCF7 xenografts treated or not with Tam (Obj: X40). The scale bar is 50 μM. Histogram quantifying the number of dots/cells and the *H*‐scores for nuclear PRMT5 and MEP50 are presented on the right. The mean ± SEM of 7 tumors is shown. *P*‐values were determined using a Student's *t*‐test.ERα methylation SDMA, PRMT5 and MEP50 expression were studied as in (A) on a Tam‐resistant formalin‐fixed PDX model HBCx‐34 Tam R treated or not with Tam. ERα methylation SDMA was analyzed as described above and the *H*‐score for nuclear PRMT5 and MEP50 was evaluated as in (A). The scale bar is 50 μM. The mean ± SEM of 3 tumors is shown. *P*‐values were determined using a Student's *t*‐test.ERα methylation SDMA, PRMT5 and MEP50 expression were studied as in (A) on a Tam‐resistant formalin‐fixed the PDX model HBCx‐22 Tam R treated or not with Tam. ERα methylation SDMA was analyzed as described above and the *H*‐score for nuclear PRMT5 and MEP50 was evaluated as in (A). The scale bar is 50 μM. The mean ± SEM of 7 tumors is shown. Source data are available online for this figure.

*In vivo*, we initially validated our SDMA‐based ERα methylation model by showing the lack of methylation in ERα‐negative tumor samples from a patient‐derived xenograft (PDX) model of BC and in a paraffin‐embedded section of fresh breast tumor treated with G595 (Appendix Fig S4A and B). In parallel, we observed a significant increase in methylation in a PDX model engrafted in ovariectomized mice, (Appendix Fig S4C), reinforcing our *in cellulo* results showing that E_2_ impedes ERα methylation (Fig 2B–D). We then used fixed tumors from mice engrafted with MCF7 cells treated or not with Tam, and confirmed that Tam induced a significant increase in ERα methylation (Fig 6A). Interestingly, although PRMT5 and MEP50 were exclusively localized in the cytoplasm of cells in untreated mice, Tam triggered a massive nuclear expression of both proteins as evidenced by IHC staining. Inversely, PRMT5 and MEP50 displayed no nuclear translocation in two Tam‐resistant PDX models treated or not with Tam (Cottu *et al*, 2014) (Fig 6B and C). In addition, in these two resistant models, Tam treatment did not increase ERα methylation.

In conclusion, our results strongly suggest that the presence of PRMT5 in the nucleus may participate in the anti‐proliferative effect of Tam in BC.

### Relevance of ERα/SDMA methylation model to tumors in our validation cohort

**Figure 7:**
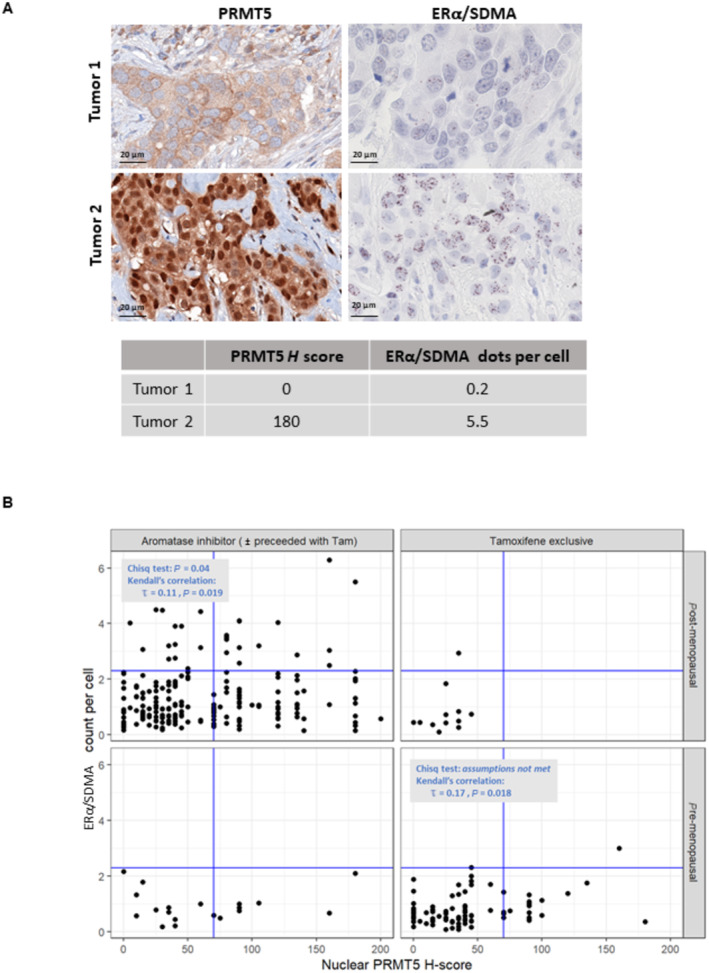
Analysis of ERα/SDMA expression in the Validation cohort For each tumor, we analyzed the level of ERα SDMA methylation by PLA. The *H*‐score for nuclear PRMT5 expression and the number of dots/cell are listed under the figures. The scale bar is 20 μm.The correlation between nuclear PRMT5, *H*‐score and ERα/SDMA expression according to hormonotherapy received. Blue lines indicate the cutoffs of 70 and 2.3 defined for PRMT5 *H*‐score and ERα/SDMA counts, respectively, used for the Chi‐squared tests. Rank correlation analysis using Kendall's method was also performed. Source data are available online for this figure.

Lastly, we evaluated ERα/SDMA methylation by PLA in the Validation cohort, and observed two types of responses (Fig 7A, and signal quantification in the table underneath). Tumor 1 was representative of tumors not expressing nuclear PRMT5 and displayed no ERα/SDMA methylation, whereas Tumor 2 was representative of tumors with high nuclear PRMT5 staining and had a high nuclear ERα/SDMA expression. We found that high levels of ERα/SDMA were mainly observed for post‐menopausal patients (Appendix Table S5). We then performed a correlation analysis between ERα/SDMA expression and nuclear PRMT5 staining, and found a weak correlation (Fig 7B). When we focused on the 16 premenopausal patients of the Tam exclusive group expressing a *H*‐score for nuclear PRMT5 > 70, none of them relapsed from BC with a long‐term follow‐up, though some patients presented poor clinical or histological parameters, such as stage T4 or grade 3. Of note, only one tumor expressed a high level of ERα/SDMA (number of dots/cell = 3) (Appendix Table S1), confirming that PRMT5 appears to be the best predictive marker of Tam sensitivity.

## Discussion

Tamoxifen (Tam), which targets ERα, is one of the preferred treatment options in the adjuvant setting for pre‐menopausal women with ERα‐positive BC. However, resistance to this treatment associated with disease relapse is a major clinical issue (Rondón‐Lagos *et al*, 2016). Despite numerous investigations on these resistance mechanisms, sensitivity to Tam is currently only based on ERα expression, and novel biomarkers are thus needed to select patients for alternative adjuvant endocrine therapies. In the present study, we showed in two independent cohorts of BC specimen that a high expression of PRMT5 in the nucleus of tumor cells was associated with a prolonged survival for patients treated with Tam but not with AI. We also highlighted a novel mechanism of ERα regulation through arginine methylation by PRMT5. Indeed, our finding introduces a new paradigm whereby nuclear PRMT5 controls the recruitment of transcriptional repressors to the receptor upon Tam treatment to achieve anti‐tumoral response. Therefore, our results identified PRMT5 as a potential predictive marker of Tam sensitivity for BC patients that could be used to better define the endocrine treatment strategy.

PRMT5 is the major type II PRMT and is implicated in a growing number of processes, including transcriptional regulation, cell signaling and DNA repair by methylating a large number of substrates (Motolani *et al*, 2021) (Stopa *et al*, 2015). Among them, PRMT5 has already been shown to methylate members of the nuclear receptor family (Malbeteau *et al*, 2022). Indeed, our team recently showed that upon dexamethasone treatment, PRMT5 triggers methylation of the glucocorticoid receptor (GR), although the functional consequences have not yet been deciphered (Poulard *et al*, 2020). In addition, PRMT5 methylates the androgen receptor on the arginine 761 residue located in the ligand‐binding domain inhibiting its transcriptional activity (Mounir *et al*, 2016). Here, we identified that PRMT5 methylates ERα in its DNA‐binding domain (DBD). As this domain is highly conserved among nuclear receptors, it is likely that PRMT5 methylates other members of the family in this domain to modulate their transcriptional activity. We were unable to identify the target arginine residue among the six arginine residues present in this region, even after several attempts, likely because the DBD is highly structured and rich in basic residues. Indeed proteases such as trypsin generate small peptides that are not detected by mass spectrometry analysis. A similar problem linked to the detection of a methylation event in this region was reported by Zhang *et al* (2013), who found it challenging to measure the methylation of K266 of ERα by SMYD2.

To circumvent this problem, we set up a PLA experiment to measure ERα methylation in fixed cells or fixed tissues as previously described for GR (Poulard *et al*, 2020, 2022). This technique allowed us to measure and localize ERα methylation *in vitro* and *in vivo*.

As PRMT5 was reported to have oncogenic properties, several PRMT5 inhibitors have been developed as potential therapeutic strategies to treat diverse cancers; three agents are currently being tested in human clinical trials. For example, GSK3326595 is currently being assessed in phase‐I trials for solid tumors and non‐Hodgkin lymphoma. Phase‐II trials for BC and acute myeloid leukemia are also ongoing (Wu *et al*, 2021). However, here we confirmed that nuclear PRMT5 may participate in the antitumoral response of Tam. Indeed, we clearly demonstrated that upon Tam treatment, PRMT5‐induced methylation of ERα is a prerequisite for its binding to a large number of proteins among which 35 out of 147 are involved in the repression of transcription (Fig EV5), highlighting that this mechanism is important, albeit other functions need to be explored. Among new ERα transcriptional repressors, we particularly studied SIRT2, and SAP18. Even if they have so far not been associated with ERα transcription, they are known to regulate chromatin compaction and then participate in transcriptional repression. The histone deacetylase SIRT2 repressed NEDD4 gene expression by directly binding to the NEDD4 gene core promoter and deacetylating histone H4 lysine 16 (Liu *et al*, 2013). SAP18 for Sim3A‐associated protein and HDAC1 induces histone deacetylation in the ZEB1 promoter and chromatin remodeling to achieve transcriptional repression (Wu *et al*, 2018). Although, we have no information regarding the direct interaction between ERα and these newly identified interactors, it is well‐known that SMRT binds directly to ERα. (Varlakhanova *et al*, 2010). The authors reported that ERα recruits SMRT through an unusual mode of interaction involving several contact surfaces located at the N‐ and C‐terminal domains of SMRT that bind the DBD of ERα. *In vitro*, Tam had no effect on the interaction, however *in vivo*, Tam increases ERα/SMRT interaction. As SMRT does not possess a TUDOR domain recognizing methylated arginine residues and based on our results, we can hypothesize that in a cellular context, a binder of ERα could impede the binding of SMRT. In the presence of Tam, the methylation of ERα by PRMT5 in the DBD may displace this binding, allowing the recruitment of SMRT and the subsequent blocking of transcription.

Based on our results, we can anticipate that deciphering the mechanisms involved in PRMT5 shuttling between the cytoplasm and the nucleus is of great interest. However, little information is available at present. So far, the main localization of PRMT5 in the cytoplasm of cells is currently explained by the presence of three NES and the lack of NLS (Gu *et al*, 2012). MEP50 its main regulator follows subcellular localization of PRMT5, though these authors reported that PRMT5 may be the instigator of this localization (Gu *et al*, 2012). In mouse primordial germ cells, PRMT5 shuttles between the nucleus and cytoplasm with the transcriptional repressor BLIMP‐1 (Ancelin *et al*, 2006). In osteosarcoma cells, PRMT5 is relocalized in the nucleus by the transcription factor SNAIL and its corepressor AJUBA (Hou *et al*, 2008). More recently, Akt kinase activity has shown to be involved in PRMT5 export from the nucleus in lung cancer cells, although the mechanism remains unknown (Liu *et al*, 2021). Identifying partners of PRMT5 responsible for its binding to importins to enter the nucleus in BC cells would be a major challenge in the future, particularly because this dual localization is associated with a dual function. Its presence in the cytoplasm is associated with oncogenic properties by methylating and activating the enzymatic activity of Akt, although its expression in the nucleus is associated with a better prognosis (Lattouf *et al*, 2019). Based on our results, we know that the enzymatic activity of PRMT5 is required for its presence in the nucleus of tumor cells, suggesting that the methylation of key substrates is required for the regulation of its shuttling between the cytoplasm and the nucleus. This phenomenon was also observed for the splice variant PRMT1v2 for which enzymatic activity was required for its nucleo‐cytoplasmic shuttling (Herrmann & Fackelmayer, 2009).

Our analysis of PRMT5 expression in two cohorts of BC patients clearly showed that its presence in abundance in the nucleus of tumor cells is associated with an increase in survival in patients treated with Tam. This is particularly striking for the Validation cohort, where none of the 16 patients expressing a high level of nuclear PRMT5 relapsed. Interestingly, some of them presented poor clinical characteristics and yet responded to Tam, highlighting nuclear PRMT5 as an independent biomarker of BC development. However, among the 16 patients, only one showed a strong ERα/SDMA expression at diagnosis. By analyzing our results in more depth, we saw that among the 33 patients with luminal tumors expressing a high level of ERα/SDMA, only three were treated with Tam, the others being treated with AI. As pre‐menopausal patients are treated with Tam and estrogens negatively regulate ERα methylation SDMA *in vitro* and *in vivo* (Fig 2A and Appendix Fig S4B), we can hypothesize that the low level of ERα SDMA methylation for these patients relies on an estrogen negative action. In conclusion, at diagnosis only nuclear PRMT5 is a predictive biomarker for Tam response. However, from our *in vivo* experiments, we can speculate that for the tumors that respond to Tam, PRMT5 and MEP50 nuclear translocation associated with an increase ERα/SDMA will participate in the anti‐tumoral properties of this anti‐estrogen.

**Figure 8:**
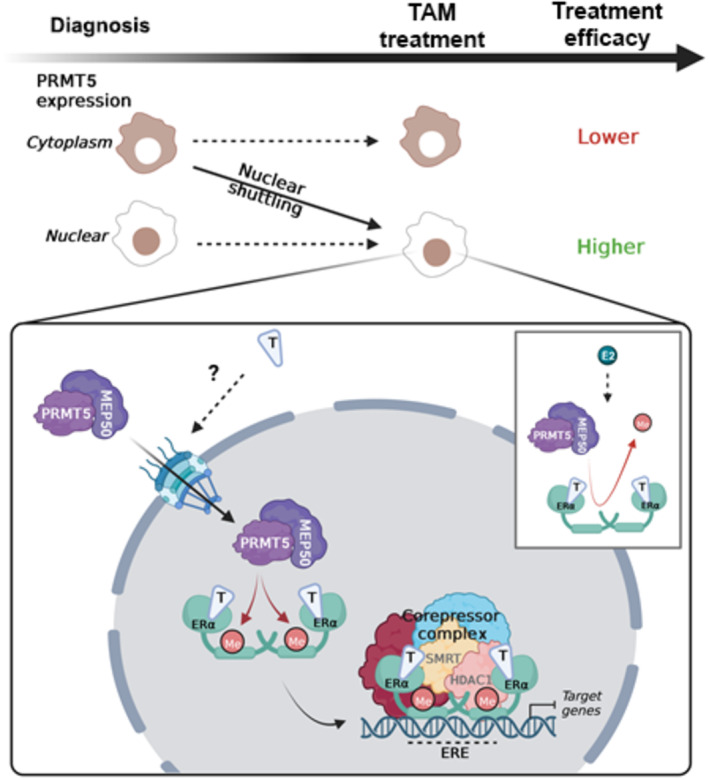
Predictive value of PRMT5 for response to Tam relies on its nuclear localization Model recapitulating our working hypothesis and results obtained in the present study. At diagnosis, the presence of a high PRMT5 expression in the nucleus of tumor cells is associated with sensitivity to Tam. Upon treatment, Tam triggers PRMT5 nuclear translocation in some tumors that become Tam‐sensitive. Mechanistically, PRMT5 methylates ERα, a key event for the binding of transcriptional corepressors. Conversely, the insert shows that E_2_ exerts the opposite effect by disrupting ERα/PRMT5 interaction and subsequently decreasing ERα methylation. Created with BioRender.com (agreement number GU24ZV3V2K).

In summary, our study identifies nuclear PRMT5 as a novel regulator of ERα involved in Tam sensitivity in ERα^+^ breast cancers. Mechanistically, upon Tam treatment the presence of PRMT5 in the nucleus triggers ERα methylation, a prerequisite for the recruitment of transcriptional repressors to ERα, impeding transcription and cell proliferation (Fig 8). Our findings shed light on a new biomarker predictive of Tam sensitivity, and deciphering mechanisms regulating the maintenance of PRMT5 in the nucleus may offer new therapeutic options. It would be of interest to investigate whether this phenomenon could be extended to other emerging SERMs currently under clinical trial (Hernando *et al*, 2021).

## Materials and Methods

### Cell culture and transfections

MCF7 cells were cultured with specific medium. The cells were purchased from ATCC and were routinely tested for mycoplasma contamination.

When stated, the cells were treated for different times with 4‐hydroxytamoxifen (Tam) (1 μM) (Sigma) or with the PRMT5 inhibitor GSK3326595 (abbreviated as G595) (Medchem express) (0.5 μM 72 h before Tam treatment).

SMART‐pool siRNAs (listed in the Appendix Table S1) were transfected into MCF7 cells using lipofectamine siRNAi max (Invitrogen) according to the manufacturer's protocol. After 48 h of transfection, proteins were analyzed.

### Human breast cancer sample collection

Early BC samples from patients treated at the Léon Bérard Cancer Center (Lyon, France) were analyzed. The activities of the biological resource center (BRC) of the CLB (n° BB‐0033‐00050), namely biological material collection and storage, are regulated by the Ministry of Research (DC‐2008‐99 and AC‐2019‐3426). Samples were collected in the context of patient diagnosis, and parts that were not used for diagnosis were reassigned to research if patients were not opposed to it (the information notice was transmitted to each patient, and written informed consent was obtained from each patient). The experiments conformed to the principles set out in the WMA Declaration of Helsinki and the Department of Health and Human Services Belmont Report. This study was approved by the ethics review board of the CLB (N° CMT2020‐16). The quality of the BRC is certified AFNOR NFS96900 (N° 2009/35884.2) and ISO 9001 (Certification N° 2013/56348.2).

In our study, patients were divided in two cohorts, named Discovery and Validation cohort. The Discovery cohort gathered data from patients diagnosed between 2001 and 2003; the Validation cohort encompassed data from patients diagnosed between 2007 and 2008 (see Flowchart in Fig EV1). Only patients diagnosed with Luminal breast cancer were included in the study (i.e., TNBC or HER2‐enriched patients were excluded). Both Discovery and Validation cohorts are retrospective observational cohorts of breast cancer patients, constituted from the breast cancer database of our institution. Accordingly, study protocol did not include any randomization or blinding treatment administration plan. Clinical and biological data, as well as outcomes were available from the regularly updated institutional database. Two groups of patients were considered: patients who received exclusively Tam treatment in the adjuvant setting, named “Tam exclusive” group, and patients who were treated with adjuvant AI and a possible previous exposure to Tam, named “AI ± Tam.”

### Patient‐derived xenograft (PDX) tumors

The tamoxifen‐resistant PDX models of ERα^+^ breast cancer HBCx‐22 TamR and HBCx‐34 TamR were established from xenografts that showed acquired resistance to tamoxifen treatment *in vivo*, as previously described (Cottu *et al*, 2014). Tumor samples from untreated and tamoxifen‐treated mice (for 110 days) were used to investigate PRMT5 and MEP50 expression by IHC analysis.

### Cell line‐derived xenograft tumors

Fixed tumors were a gift from Dr J. Carroll (Nagarajan *et al*, 2020). Subcutaneous xenografts of MCF7 cells were conceived by implanting 10^5^ cells in 50% growth medium and 50% matrigel (BD Biosciences), in the right flank of 8‐week‐old female NSG mice. The mice were also implanted subcutaneously with 90‐day slow release 17β‐estradiol (0.72 mg per pellet) hormone pellets (Innovative Research of America) into the left flank. After 4 weeks, tumors were randomized and included in the study when the average tumor volume reached 100–150 mm^3^. Mice were given 20 mg/kg Tam intraperitoneally 6 days/week. Tissues were processed and embedded in paraffin for histological assessment.

### *Ex vivo* assays

A fresh human mammary sample was obtained from a chemotherapy‐naive patient with invasive carcinoma after surgical resection at the CLB, under the responsibility of BB‐0033‐00050.

The tumor was cut into thin slices of 250 μm using a vibratome (HM 650 V Microm) and incubated for 48 h with or without 0.5 μM of G595. Slices were then fixed in 4% paraformaldehyde and paraffin embedded. Sections (4 μm) were then cut for standard histological analysis assessed by Hematoxylin phloxin saffron (HPS) staining and immunochemistry analysis using the PRMT5 antibody.

### Antibodies

All antibodies are listed in the Appendix Table S2.

### Proliferation studies

4 × 10^3^ cells seeded onto a 96‐well plate were plated 5 h before incubation with different molecules (Tam or ethanol). Images were acquired using an IncuCyte ZOOM over 7 days, and cell proliferation was measured as the percentage of cell density observed over this time‐course. Results are represented as graphs indicating the rate of proliferation over time, extrapolated from at least three independent experiments, each performed in triplicate.

### Subcellular fractionation

MCF‐7 cells were washed with PBS and fractionated into cytoplasmic and nuclear extracts using the “NE‐PER nuclear and cytoplasmic extraction kit” from Thermo Scientific following the manufacturer's guidelines.

### Immunoprecipitation and Western blot analysis

Cells were lysed using RIPA buffer (50 mM Tris HCl, pH 8.0, 150 mM NaCl, 1 mM EDTA, 1% NP‐40 and 0.25% deoxycholate) supplemented with protease inhibitor tablets (Roche Molecular Biochemicals) and phosphatase inhibitors (1 mM NaF, 1 mM Na_3_VO_4_ and 1 mM β‐glycerophosphate). Protein extracts were incubated with primary antibodies overnight at 4°C on a shaker. Protein G or A‐Agarose beads were added, and the mix was incubated for 2 h at 4°C. The immunoprecipitated proteins were separated by sodium dodecyl sulfate‐polyacrylamide gel electrophoresis (SDS‐PAGE) and analyzed by Western blot, then visualized by electrochemiluminescence (ECL, Roche Molecular Biochemicals).

### RNA extraction and real‐time RT‐qPCR analysis

Total RNA (1 μg) was extracted and purified using TRI‐Reagent (Sigma‐Aldrich, USA), prior to being reverse‐transcribed using using 1 μg total RNA as a template using Superscript III (Invitrogen, USA) protocol. Real time PCR was performed on a BioRad CFX Real‐Time PCR system using SYBR green supermic (BioRad). mRNA levels were normalized against the expression of 28S ribosomal mRNA as a reference. Primer sequences are listed in the Appendix Table S3.

### Chromatin immunoprecipitation

Chromatin immunoprecipitation experiments were performed according to the manufacturer's protocol (SingleChIP enzymatic chromatin IP Kit ‐ Cell signaling) with antibodies against ERα, SMRT and IgG. Results are expressed relative to the signal obtained with chromatin input. Primer sequences are indicated in the Appendix Table S3.

### *In vitro* methylation assays

The PRMT5/MEP50 complex (purchased from Sigma Aldrich) was incubated with the different domains of ERα fused to GST as already described (Le Romancer *et al*, 2008) in the presence of S‐adenosyl‐L [methyl‐^3^H] methionine ([^3^H] SAM 85 Ci/mmol from a 10.4 mM stock solution in dilute HCl/ethanol 9/1 [pH 2.0–2.5]; Perkin Elmer) for 90 min at 30°C. Methylation reactions were quenched by adding Laemmli sample buffer, heated at 95°C for 5 min, and separated by SDS–PAGE. Following electrophoresis, gels were soaked in Amplify reagent (Sigma) according to the manufacturer's instructions and visualized by autoradiography.

### Proximity ligation assay, image acquisition and analysis

Protein ligation assay (PLA) exposes protein/protein interactions *in situ* (Söderberg *et al*, 2006). Briefly, cells were seeded and fixed with cold methanol. After saturation, the different couples of primary antibodies were incubated for 1 h at 37°C. The PLA probes consisting of secondary antibodies conjugated with complementary oligonucleotides were incubated for 1 h at 37°C. The amplification step followed the ligation of nucleotides for 100 min at 37°C. Samples were subsequently analyzed under fluorescence microscopy.

The hybridized fluorescent slides were viewed under a Nikon Eclipse Ni microscope. Images were acquired under identical conditions at ×40 magnification. Image acquisition was performed by imaging DAPI staining at a fixed Z Position while a Z stack of ±5 μm at 1 μm intervals was carried out. The final image was stacked to a single level before further quantification. On each sample, at least one hundred cells were counted. Analysis and quantification of these samples were performed without randomization/blinding using the Image J software (free access). PLA dots were quantified on 8‐bit images using the “Analyze Particles” command, while cells were counted using the cell counter plugin as previously described (Poulard *et al*, 2020).

### Glutathione transferase (GST) pull‐down assay

An ERα‐expressing plasmid was transcribed and translated *in vitro* using T7‐coupled reticulocyte lysate. GST and GST‐PRMT5 proteins were incubated with labeled proteins in 200 μl of binding buffer (Tris 20 mM pH 7.4, NaCl 0.1 M, EDTA 1 mM, glycerol 10%, Igepal 0.25% with 1 mM DTT and 1% milk) for 2 h at room temperature. After washing, bound proteins were separated by SDS–PAGE and visualized by Western blot.

### Immunohistochemistry staining

Formalin‐fixed paraffin embedded tumor tissues were used for analysis. The pathologist selected representative areas from breast invasive carcinomas. Triplicates from each tumor were inserted into TMA blocks which contained 40 tumors each. After deparaffinization and rehydration, tissue sections were boiled in 10 mM citrate buffer pH 8.0 at 95°C for 40 min. The slides were then incubated in 5% hydrogen peroxide in sterile water to block the activity of endogenous peroxidases. The slides were then incubated at 37°C for 1 h with the primary antibodies. The slides were subsequently incubated with a biotinylated secondary antibody bound to a streptavidin peroxidase conjugate (Envision Flex kit Ref: K800021‐2, Dako). Bound antibodies were detected by adding the substrate 3,3‐diamino benzidine. Sections were counterstained with hematoxylin.

For scoring purposes, the intensity of staining in malignant cells was divided into four groups (0: no staining, 1: weak staining, 1.5: moderate staining, 2: strong staining) and the percentage of stained cells was reported separately. Both intensity and percentage scores were then multiplied to obtain a single *H*‐score.

### BIFC screening

#### Cell lines

MCF7 cells were purchased from the European Collection of Authenticated Cell Cultures (ECACC) and cultured in Dulbecco's modified Eagle's medium (DMEM‐GlutaMAX, Gibco, Life Technologies) supplemented with 10% fetal bovine serum (FBS), 1% (v/v) penicillin–streptomycin (5,000 U penicillin 5 mg streptomycin/ml). These cells were transformed to express a bank composed of 1,812 pre‐selected open reading frames (ORF) (Dataset EV1) following the patent FR3052788A1 recommendation (Jia *et al*, 2023). The generated MCF7‐PCA cell line was selected and cultured using complete medium supplemented with 0.5 μg/ml of puromycin (Gibco, Life Technologies).

#### Cell‐PCA screening

Three MCF7‐PCA cell vials were thawed and passaged separately for 5 days to recover. For each screening condition and replicate 3 × 10^6^ cells were seeded in T75 flasks in complete medium supplemented with 0.5 μM G595 or DMSO. Upon reaching 80% confluency, depending on the condition, the medium was replaced with medium supplemented with 0.5 μM GSK3326595 and/or 1 μM Tamoxifen and/or DMSO/ethanol. Subsequently MCF7‐PCA cells were transfected with pFN‐ ERα. Transfections were performed using JetPrime (polyplus) following the manufacturer's instructions.

Cells containing a candidate interactor were sorted by flow cytometry using BD FACS Aria II.

Genomic DNA extraction was performed for each harvested cell pool using PureLINK genomic DNA MiniKit (Invitrogen™). Genomic DNA samples were used for next generation sequencing (PSI, IGFL, Lyon, France) based on their own proprietary protocol.

#### ERα‐positive candidate identification

The sequencing results were normalized by count per million. To reduce background noise, a threshold was then set at 300 reads. This threshold corresponds to the value in cpm of the internal control HDAC1. For the final list of interactors, only ORF superior to the threshold in all replicates of the same condition were selected (Dataset EV1).

### Statistical analysis

#### Descriptive analysis

The distribution of clinical parameters (cancer subtype, clinical, histological and immunohistochemical data) was presented separately for the Discovery and Validation cohorts, as numbers and percentages for categorical variables or as mean, standard deviation, and range for continuous variables. Nuclear PRMT5 expression level was discretized using the previously published cutoff of *H*‐score equal to 70, considering low expression as PRMT5_hscore_ ≤ 70 and high expression as PRMT5_hscore_ > 70 (Lattouf *et al*, 2019). Statistical association between expression levels and clinical parameters or biomarkers were conducted using chi‐squared or Fisher's exact test for categorial variables and Student's *t*‐test for continuous variables.

#### Survival analysis

The main outcome analyzed was disease‐free survival (DFS), defined as time elapsed from the date of diagnosis to the date of the first event or the date at last news, considering as an event either local‐ or distant‐disease progression, or death from any cause. Survival curves were drawn with the Kaplan Meier method and compared using the log‐rank test. Unadjusted and adjusted hazard ratios were estimated using Cox regression models, fitted separately for the Discovery and Validation cohorts, and combined using a fixed‐effects meta‐analysis model (Laird & Mosteller, 1990). All multivariable Cox models were adjusted on the following pre‐specified list of factors measured at diagnosis: age, BMI, menopausal status, pathological stage, grade and presence/absence of lymphovascular invasion. Test of interaction between PRMT5 expression and patient groups according to the endocrine therapy (Tam exclusive vs. AI ± Tam) was performed using a Cox model fitted on a pooled dataset (pooling Discovery and Validation data), and stratifying the survival baseline hazard rate on cohort (i.e., using a “strata(cohort)” term in the Cox model formula) (Harrell, 2001). The same approach was used to test interactions between PRMT5 expression and pathological stage at diagnosis. Ten‐year absolute survival risk estimates were estimated from a fixed‐effects meta‐analysis Cox model (combining models from Discovery and Validation cohorts), stratified according to pathological stage, PRMT5 expression, and endocrine treatment groups.

All statistical analyses were carried out using the R software (R Core Team, 2020).

This study includes no data deposited in external repositories.

## Author contributions

**Coralie Poulard:** Conceptualization; investigation; methodology; writing – review and editing. **Thuy Ha Pham:** Investigation; methodology. **Youenn Drouet:** Formal analysis; investigation; methodology; writing – review and editing. **Julien Jacquemetton:** Validation; methodology. **Ausra Surmielova:** Methodology. **Benoite Mery:** Methodology. **Loay Kassem:** Methodology. **Jonathan Reboulet:** Methodology. **Christine Lasset:** Methodology. **Isabelle Treilleux:** Investigation; methodology. **Elisabetta Marangoni:** Investigation; methodology. **Olivier Trédan:** Resources; supervision; funding acquisition; project administration; writing – review and editing. **Muriel Le Romancer:** Conceptualization; supervision; funding acquisition; writing – original draft; project administration; writing – review and editing.

## Disclosure and competing interests statement

The authors declare that they have no conflict of interest.

## Supporting information



Appendix

Expanded View Figures PDF

Dataset EV1

PDF+

Source Data for Figure 1

Source Data for Figure 2

Source Data for Figure 3

Source Data for Figure 4

Source Data for Figure 5

Source Data for Figure 6

Source Data for Figure 7

## Data Availability

This study includes no data deposited in external repositories.
