# Supplementary material for: Nuclear PRMT5 is a biomarker of sensitivity to tamoxifen in ERα + breast cancer
Source: EMBO Mol Med. 2023 Jul 17;15(8):e17248. doi: 10.15252/emmm.202217248 (PMC10405064; doi:10.15252/emmm.202217248)
Supplement: Supplementary file 1 — Appendix [file EMMM-15-e17248-s011.docx]

*Appendix data*

**Nuclear PRMT5 is a biomarker for sensitivity to tamoxifen in ERα+ breast cancer**

The appendix file includes 5 Tables and 4 Figures

**Contents**

**Appendix Table S1**. List of siRNA sequences used in the present study………………………2

**Appendix Table S2**. List and origin of the antibodies used in the current work……………….3

**Appendix Table S3.** List of primers used in the current study…………………………………4

**Appendix Table S4**. Clinico-pathological characteristics of patients at diagnosis for the Validation cohort, according to nuclear ERα/SDMA expression………………………… ….5

**Appendix Table S5.** Clinico-pathological characteristics of patients treated with Tam and with PRMT5 H-score>70…………………………………………………………………………… 7

**Appendix Figure S1**. Predicted 10-year disease-free survival probability according to PRMT5 nuclear expression, stage at diagnosis, and menopausal status, for patients treated with Tam and AI………………………………………………………………………………………………….8

**Appendix Figure S2**. Control PLA experiments for ERα/PRMT5 interactions……… …….9

**Appendix Figure S3**. Controls for ERα methylation by PLA……………………………… 10

**Appendix Figure S4.** Validation of ERα/SDMA expression in BC PDX models………… …11

**Appendix Table S1.** List of siRNA sequences used in the present study

| **siRNA** | **Reference** | **Supplier** |
| --- | --- | --- |
| si:scramble | D-001810-02-05 | Horizon Discovery |
| si-PRMT5 | L-015817-00-0005 | Horizon Discovery |
| si:ERα | L-003401-00-0005 | Horizon Discovery |
| si:MEP50 | L-006895-00-0005 | Horizon Discovery |

**Appendix Table S2.** List and origin of the antibodies used in the current work

| **Antibody** | **Company** | **Ref.** | **Species** | **WB** | **IP** | **PLA** | **IHC** |
| --- | --- | --- | --- | --- | --- | --- | --- |
| SDMA | CST | 13222S | rabbit | 1:1000 |  | 1/200 |  |
| ERα | Santa Cruz | sc-8002 | mouse |  |  | 1/200 |  |
| ERα | Sigma Aldrich | 04-820 | rabbit | 1/1000 |  |  |  |
| ERα | Thermo Fisher | MA5-13191 | mouse |  |  | 1/200  1/400 |  |
| PRMT5 | Sigma Aldrich | 07-405 | Rabbit |  | 1/200 | 1/100 |  |
| PRMT5 | CST | 79998S | Rabbit | 1/1000 |  |  |  |
| MEP50 | CST | 2823S | Rabbit | 1/1000 |  |  |  |
| SMRT | CST | 62370S | Rabbit | 1/1000 | 1/200 |  |  |
| SMRT | Thermo Fisher | PA1-843 | Rabbit |  |  | 1/500 |  |
| HDAC1 | CST | 34589T | Rabbit | 1/1000 |  | 1/800 |  |
| GAPDH | Santa Cruz | sc-47724 | mouse | 1/1000 |  |  |  |
| β-Tubulin | Sigma Aldrich | T6074 | mouse | 1/10000 |  |  |  |
| Histone H3 | CST | 4499S | rabbit | 1/1000 |  |  |  |

**Appendix Table S3.** List of primers used in the current study

| **Genes** | **Primer type** | **Sequence (5’-3’)** |
| --- | --- | --- |
| GREB1 ERE | Forward  Reverse | CAGCTGACTGTCTTCCACCA  CCACCGTTTCGTGTCTTCTT |
| TFF1 ERE | Forward  Reverse | CACCCGTGAGCCACTGT  CTGCAGAAGTGATTCATAGTGAGAGAT |
| XBP1 ERE | Forward  Reverse | ATACTTGGCAGCCTGTGACC  GGTCCACAAAGCAGGAAAAA |
| GREB1 | Forward  Reverse | CAAAGAATAACCTGTTGGCCCTGC  GACATGCCTGCGCTCTCATACTTA |
| TFF1 | Forward  Reverse | CACCATGGAGAACAAGGTGA  TGACACCAGGAAAACCACAA |
| XBP1 | Forward  Reverse | GCTGATGACGTCCCCACT  GTTGGGCATTCTGGACAACT |

**Appendix Table S4:** Clinico-pathological characteristics of patients at diagnosis for the Validation cohort, according to nuclear ERα/SDMA expression

|  | **ERα/SDMA level** | |  |  |
| --- | --- | --- | --- | --- |
|  | **Low (0-2.3) (N=300)** | **High (2.3-20) (N=33)** | **ALL (N=333)** | **p value** |
| **Age at diagnosis** |  |  |  | < 0.001 |
| Mean (SD) | 56.57 (11.96) | 65.00 (12.30) | 57.41 (12.24) |  |
| Min. – Max. | 25.00 - 88.00 | 30.00 - 86.00 | 25.00 - 88.00 |  |
| **Age at diagnosis (cat.)** |  |  |  | < 0.001 |
| ≤50 | 106 (35.3%) | 3 (9.1%) | 109 (32.7%) |  |
| [50-65[ | 130 (43.3%) | 13 (39.4%) | 143 (42.9%) |  |
| [65+ | 64 (21.3%) | 17 (51.5%) | 81 (24.3%) |  |
| **Menopausal status** |  |  |  | < 0.001 |
| Post | 192 (64.0%) | 31 (93.9%) | 223 (67.0%) |  |
| Pre | 108 (36.0%) | 2 (6.1%) | 110 (33.0%) |  |
| **BMI** |  |  |  | 0.620 |
| Missing | 9 | 1 | 10 |  |
| Mean (SD) | 24.98 (4.82) | 25.44 (5.80) | 25.03 (4.91) |  |
| Min. – Max. | 15.00 - 41.00 | 17.00 - 38.00 | 15.00 - 41.00 |  |
| **BMI (cat.)** |  |  |  | 0.011 |
| Missing | 9 | 1 | 10 |  |
| ≤18.5 | 9 (3.1%) | 4 (12.5%) | 13 (4.0%) |  |
| [18.5-25[ | 167 (57.4%) | 13 (40.6%) | 180 (55.7%) |  |
| [25 - 30[ | 78 (26.8%) | 7 (21.9%) | 85 (26.3%) |  |
| [30+ | 37 (12.7%) | 8 (25.0%) | 45 (13.9%) |  |
| **Progesterone Receptor** |  |  |  | 0.972 |
| Negative | 37 (12.3%) | 4 (12.1%) | 41 (12.3%) |  |
| Positive | 263 (87.7%) | 29 (87.9%) | 292 (87.7%) |  |
| **SBR grade** |  |  |  | 0.973 |
| I | 69 (23.0%) | 7 (21.2%) | 76 (22.8%) |  |
| II | 160 (53.3%) | 18 (54.5%) | 178 (53.5%) |  |
| III | 71 (23.7%) | 8 (24.2%) | 79 (23.7%) |  |
| **Surgery type** |  |  |  | 0.691 |
| Mastectomy | 129 (43.0%) | 13 (39.4%) | 142 (42.6%) |  |
| Tumorectomy | 171 (57.0%) | 20 (60.6%) | 191 (57.4%) |  |
| **Pathological T** |  |  |  | 0.716 |
| T1 | 165 (55.0%) | 20 (60.6%) | 185 (55.6%) |  |
| T2 | 72 (24.0%) | 8 (24.2%) | 80 (24.0%) |  |
| T3-T4 | 63 (21.0%) | 5 (15.2%) | 68 (20.4%) |  |
| **Pathological N** |  |  |  | 0.425 |
| N0 | 148 (49.3%) | 18 (54.5%) | 166 (49.8%) |  |
| N1 | 76 (25.3%) | 5 (15.2%) | 81 (24.3%) |  |
| N2-N3 | 76 (25.3%) | 10 (30.3%) | 86 (25.8%) |  |
| **Metastasis at diagnosis** |  |  |  |  |
| M0 | 300 (100.0%) | 33 (100.0%) | 333 (100.0%) |  |
| **Pathological Stage** |  |  |  | 0.979 |
| I | 101 (33.7%) | 11 (33.3%) | 112 (33.6%) |  |
| II | 86 (28.7%) | 10 (30.3%) | 96 (28.8%) |  |
| III | 113 (37.7%) | 12 (36.4%) | 125 (37.5%) |  |
| **Lymphovascular invasion** |  |  |  | 0.355 |
| No | 197 (65.7%) | 19 (57.6%) | 216 (64.9%) |  |
| Yes | 103 (34.3%) | 14 (42.4%) | 117 (35.1%) |  |
| **Chemotherapy** |  |  |  | 0.313 |
| No | 145 (48.3%) | 19 (57.6%) | 164 (49.2%) |  |
| Yes | 155 (51.7%) | 14 (42.4%) | 169 (50.8%) |  |
| **Radiotherapy** |  |  |  | 0.118 |
| No | 22 (7.3%) | 5 (15.2%) | 27 (8.1%) |  |
| Yes | 278 (92.7%) | 28 (84.8%) | 306 (91.9%) |  |
| **Hormonotherapy** |  |  |  | 0.005 |
| Missing | 6 | 1 | 7 |  |
| Aromatase inhibitor (+/- preceeded with Tam) | 196 (66.7%) | 29 (90.6%) | 225 (69.0%) |  |
| Tamoxifen exclusive | 98 (33.3%) | 3 (9.4%) | 101 (31.0%) |  |

**Appendix Table S5:** Clinico-pathological characteristics of patients treated with Tam and with nuclear PRMT5 (H-score>70)

**
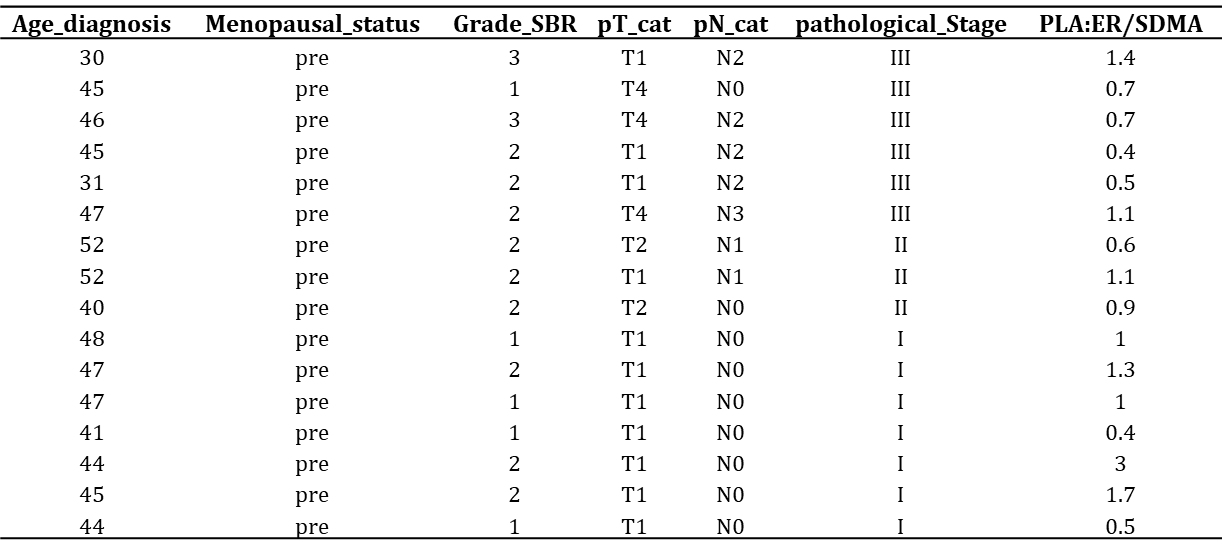
**

This table presents the clinical characteristics and ERα/SDMA for the 16 patients treated with Tam with nuclear PRMT5 (H-score>70)

**
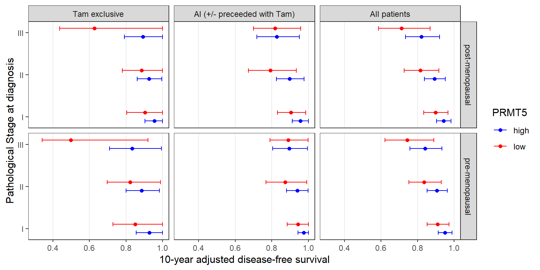
**

**Appendix Figure S1: Predicted 10-year disease-free survival probability according to PRMT5 nuclear expression, stage at diagnosis, and menopausal status, for patients treated with Tam and AI.** Horizontal bars indicate 95% confidence intervals.

**
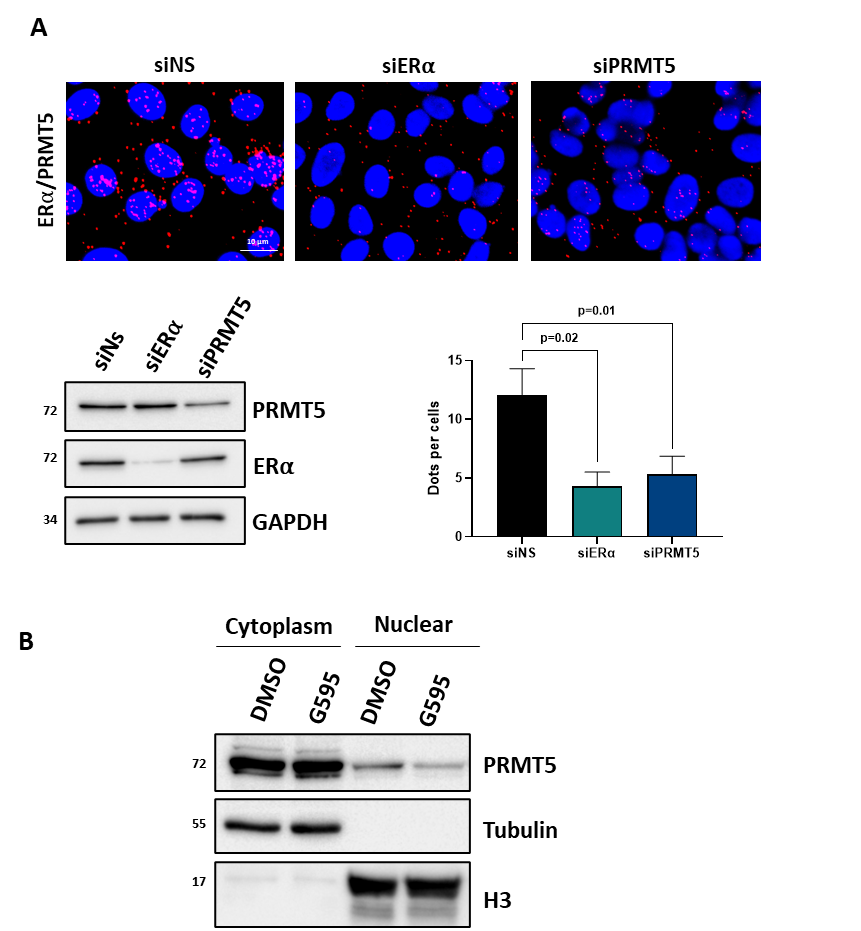
**

**Appendix Figure S2: Control PLA experiments for ERα/PRMT5 interactions.**

**A.** MCF7 cells were transfected with a non-specific siRNA (siNS) or siRNA targeting ERα, or PRMT5 for 48 h. After fixation, proximity ligation assay (PLA) experiments were performed to evaluate the interactions between ERα/PRMT5 using specific antibodies. The detected dimers are represented by red dots. The nuclei were counterstained with mounting medium containing DAPI (blue) (Obj:X60). Quantification of the number of dots per cell was performed by computer-assisted analysis as reported in the Materials and Methods section. The mean SEM of one experiment representative of three experiments is shown. The efficacy of protein inhibition was verified by Western blot analysis using the corresponding antibodies (lower panels). **B.** After G595 treatment for 72 h, MCF7 cells were fractionated and each fraction was assessed for PRMT5, tubulin and Histone H3 expression by western blotting.

**
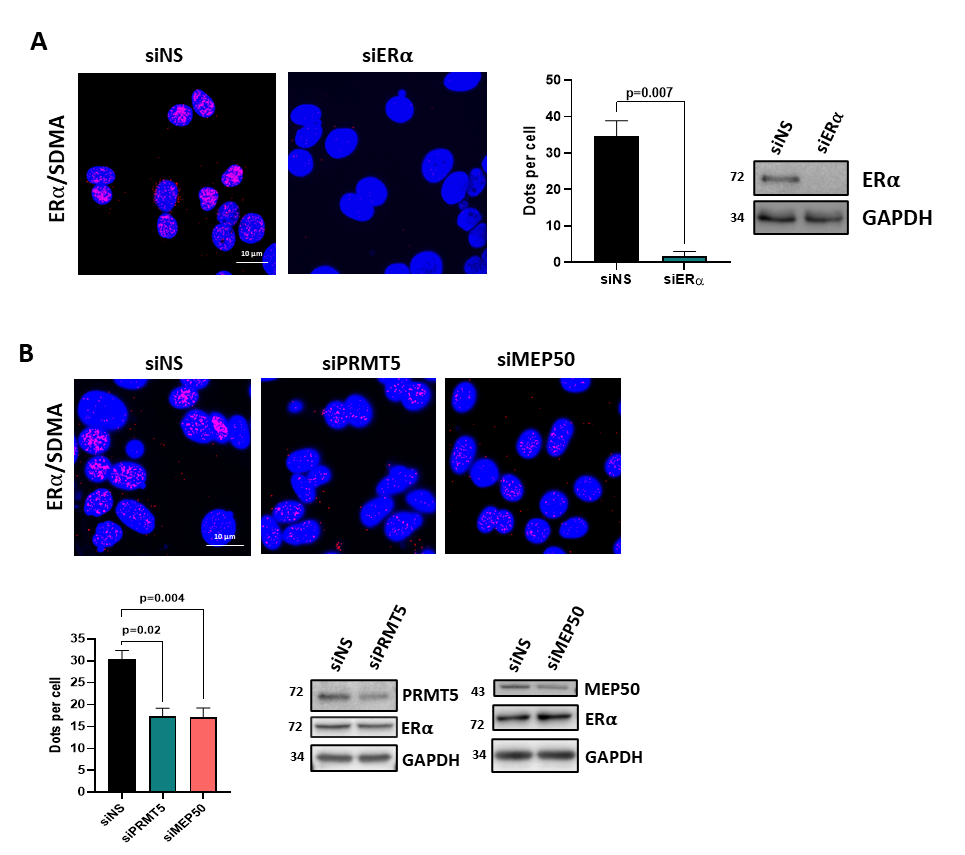
**

**Appendix Figure S3: Controls for ERα methylation by PLA.**

**A.** MCF7 cells were transfected with siNS or siRNAs targeting ERα. After fixation, proximity ligation assay (PLA) was performed to evaluate ERα methylation using an anti-ERα antibody and a pan SDMA antibody. The detected dimers are represented by red dots. The nuclei were counterstained with DAPI (blue) (Obj.: X60). Quantification of the number of dots per cell was performed by computer-assisted analysis as reported in the Materials and Methods section. The mean ± SEM of one experiment representative of three experiments is shown. The efficacy of protein inhibition was verified by Western blotting using the corresponding antibodies.

**B.** The same experiment was performed with siRNA targeting PRMT5 or MEP50. The *p*-value was determined using the Student t-test.

**
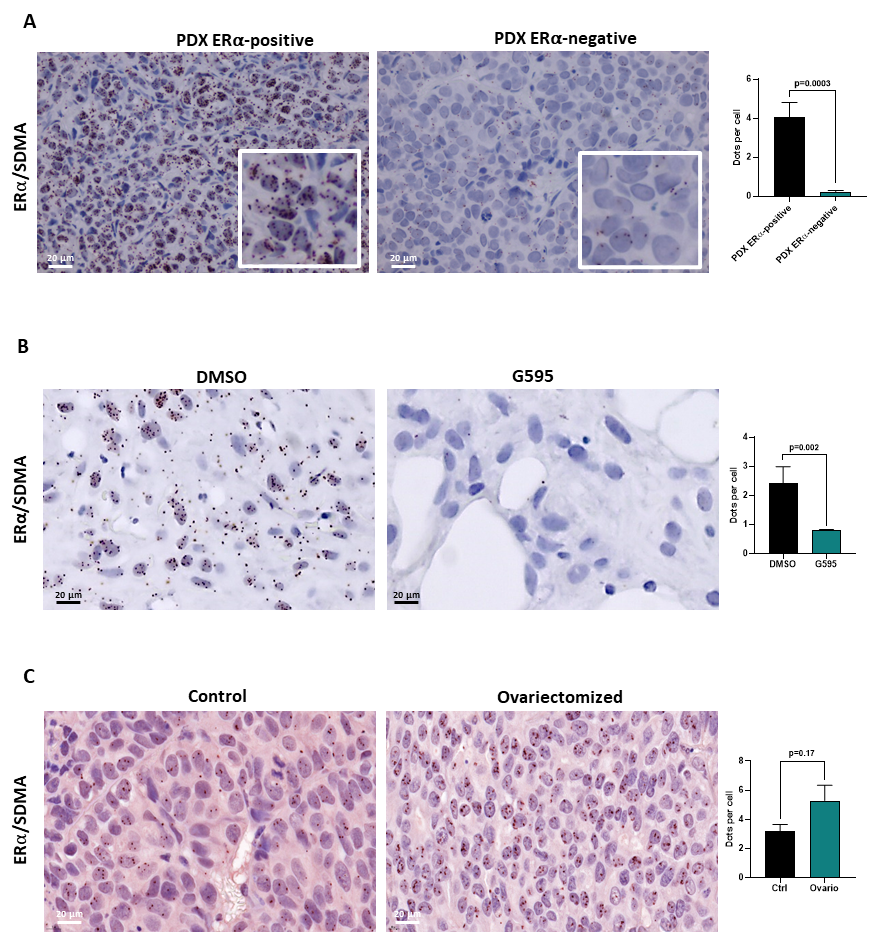
**

**Appendix Figure S4**: **Validation of ERα/SDMA expression in vivo**.

**A.** Tumors from two PDX models of breast cancer were embedded in paraffin. A bright-field PLA was performed to study ERα methylation in the two PDX models. (X40 magnification). In the right panel, the number of dots per tumor cell were quantified as described in the Materials and Methods section. The *p*-value was determined using a Student t-test. **B.** Paraffin-embedded sections from a fresh tumor were incubated with or without G595 and ERα/SDMA was assessed by PLA (Obj: X40). H-scores for nuclear PRMT5 expression are also presented **C.** ERα/SDMA expression was studied in the PDX model HBCx-34 TamR in normal (same picture as Figure 6B) or ovariectomized mice. In the right panel, the number of dots per tumor cell were quantified as in A. The *p*-value was determined using a Student t-test.
