# Supplementary material for: Nuclear PRMT5 is a biomarker of sensitivity to tamoxifen in ERα + breast cancer
Source: EMBO Mol Med. 2023 Jul 17;15(8):e17248. doi: 10.15252/emmm.202217248 (PMC10405064; doi:10.15252/emmm.202217248)
Supplement: Supplementary file 2 — Expanded View Figures PDF [file EMMM-15-e17248-s001.pdf]

Expanded View Figures

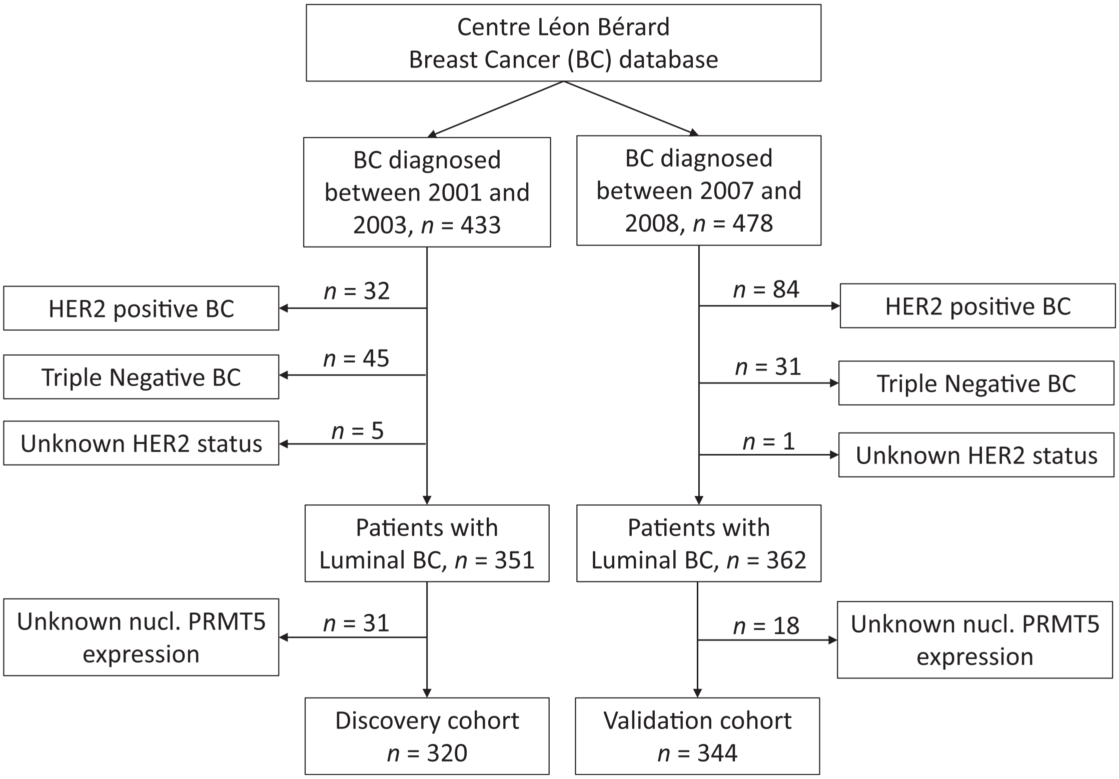

Figure EV1. Flowchart describing the Discovery and Validation cohorts.

Figure EV2. Study of ERα/SDMA in ERα-positive BC cells treated with tamoxifen.

A MCF7, Cama-1 and ZR-75 and T47D cells were treated with Tam (1 μM) or ethanol (EtOH). After fixation, proximity ligation assay (PLA) was performed to evaluate ERα methylation using specific antibodies. The detected dimers are represented by red dots. The nuclei were counterstained with DAPI (blue) (Obj: X60) (left panel).

B Quantification of the number of dots per cell was performed by computer-assisted analysis as reported in the Materials and Methods section. The mean ± SEM of one experiment representative of three experiments is shown.

C The four cell lines were plated onto 96-well plates and treated with Tam (1 μM) or ethanol (EtOH) and proliferation was measured in real time using the IncuCyte technology. Image acquisition was conducted every hour using the IncuCyte software, which calculates the percentage of cell confluency as a function of time over 7 days. The results are represented as graphs showing the rate of proliferation every 24 h. The mean ± SD of one experiment representative of three experiments is shown.

D ERα, PRMT5 and Tubulin expression were assessed by Western blotting in the four ERα-positive BC cells.

© 2023 The Authors

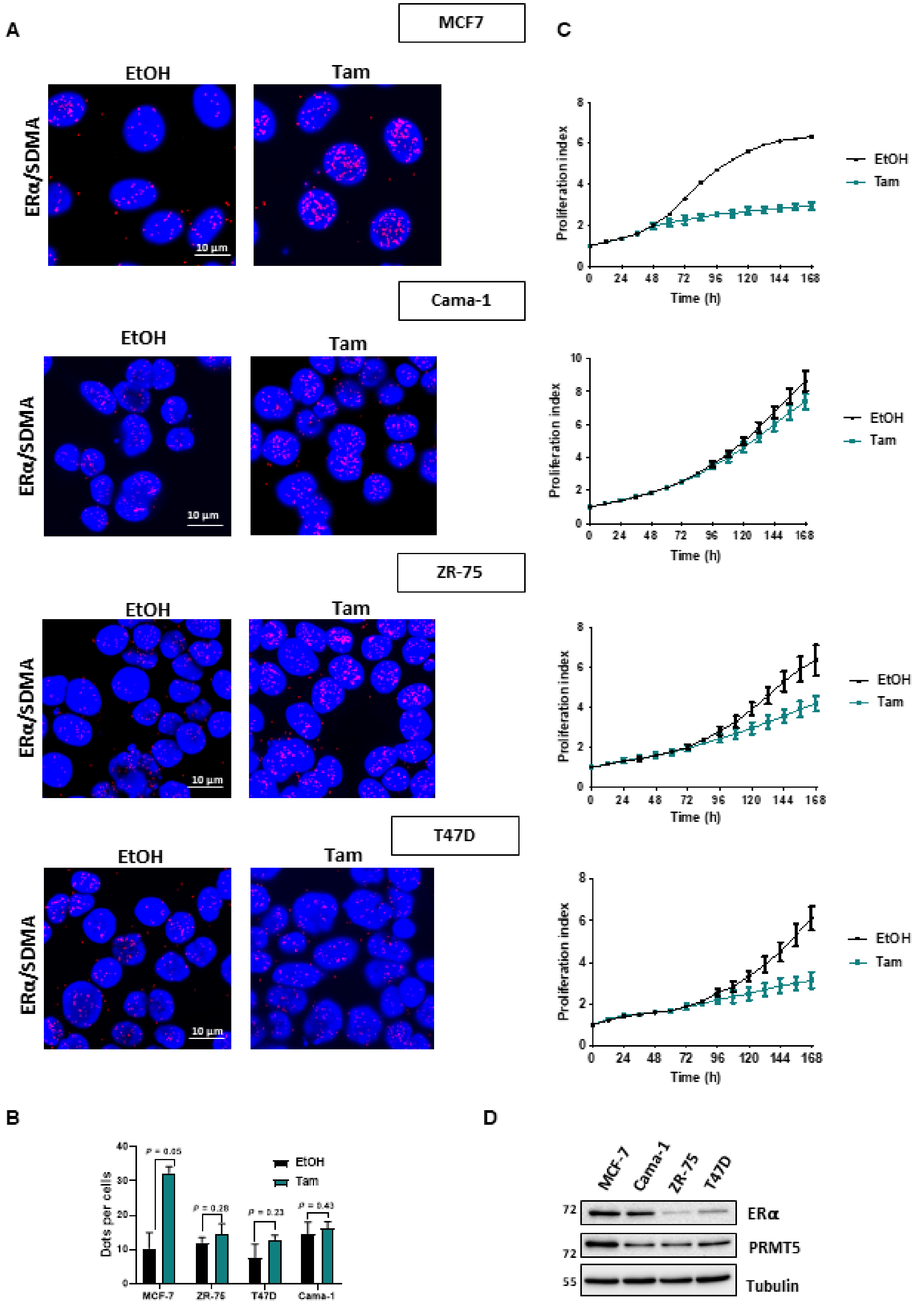

Figure EV2.

© 2023 The Authors

Figure EV3. Study of ERα/SMRT interaction in MCF7 cells.

A, B MCF7 cells were transfected with siNS or siRNAs targeting ERα or siSMRT. After fixation, PLA was performed to evaluate ERα/SMRT interactions using specific antibodies (Obj: X60). Quantification of the number of dots per cell was performed as described above. The efficacy of protein inhibition was verified by Western blotting using the corresponding antibodies.

C MCF7 were treated with Tam 1 μM for 6 h in the presence or absence of G595. Cell extracts were then used to immunoprecipitate SMRT. The presence of SMRT and ERα in the inputs were studied by Western blotting together with SDMA and GAPDH.

D MCF7 cells were transfected with siNS or siRNAs targeting PRMT5 or MEP50. After siRNA transfection and fixation, PLA was performed to evaluate ERα/SMRT interactions using specific antibodies (Obj: X60). Quantification of the number of dots per cell was performed as described above. The efficacy of protein inhibition was verified by western blotting using the corresponding antibodies. The *P*-value was determined using a Student *t*-test.

© 2023 The Authors

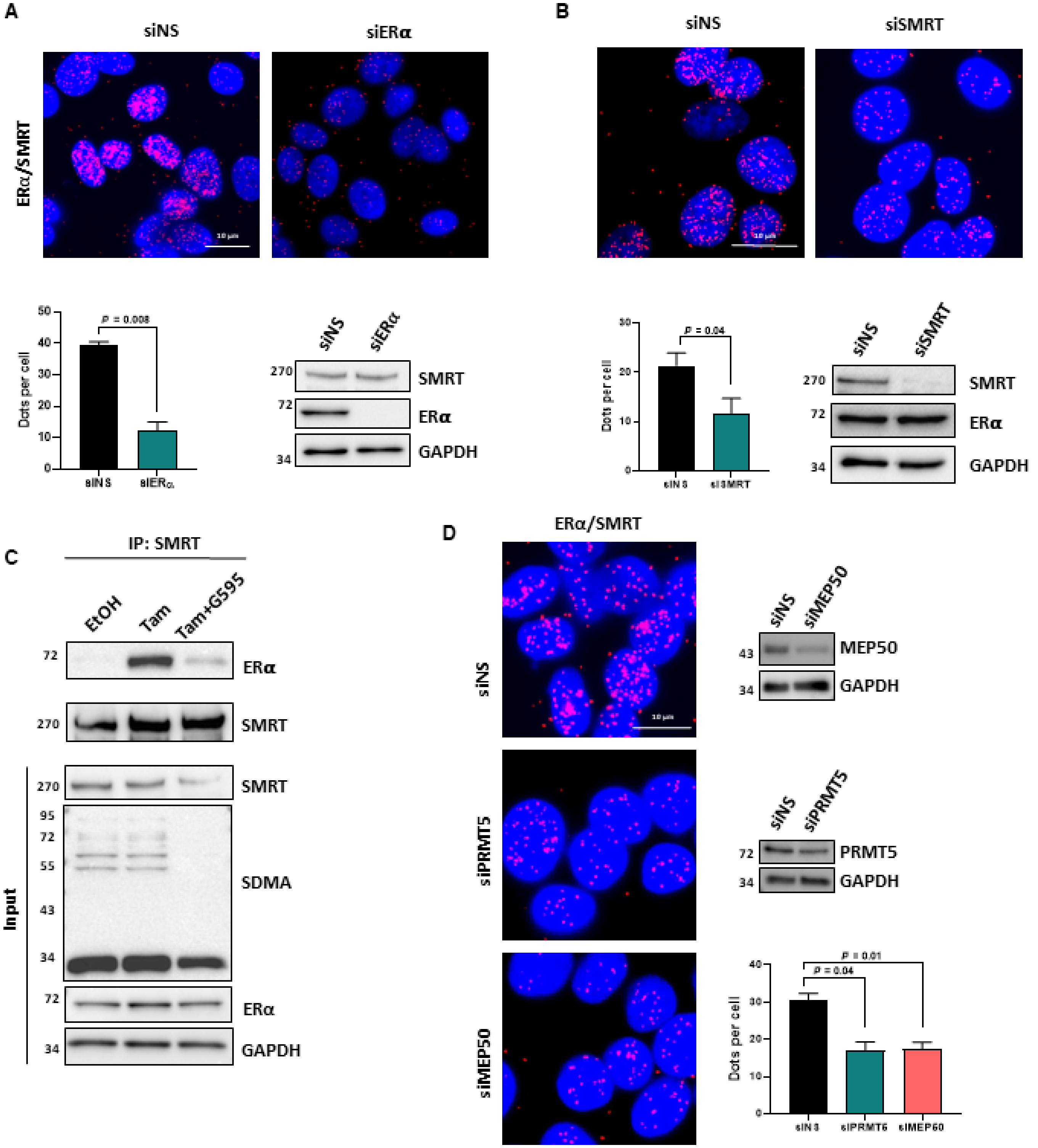

Figure EV3.

 © 2023 The Authors

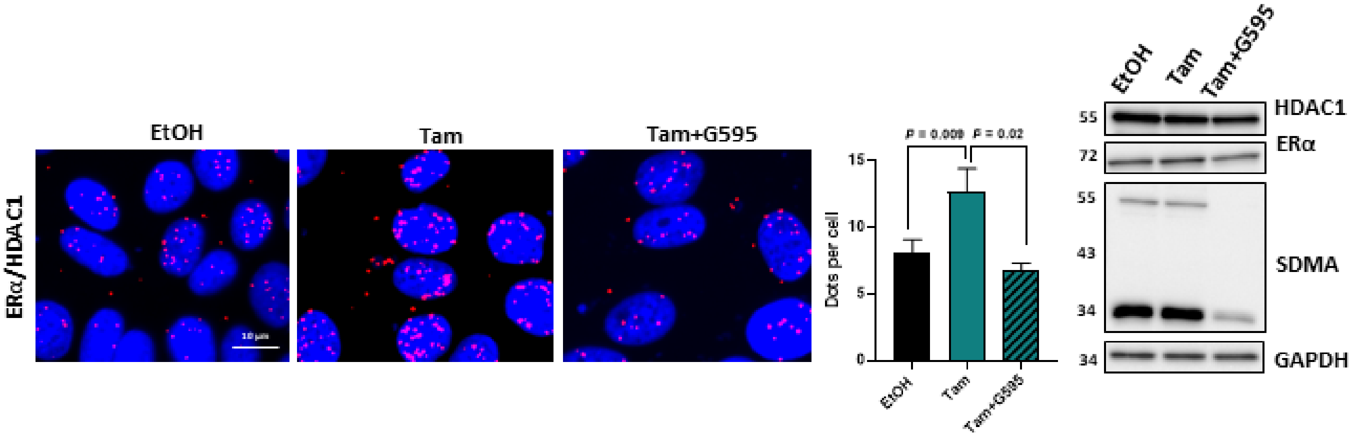

Figure EV4. Detection of ERα/HDAC1 interactions in MCF7 cells.

MCF7 were treated with Tam 1 μM for 6 h in the presence or absence of G595, the cells were then fixed and PLA was performed to study ERα/HDAC1 interactions. Quantification of the number of dots per cell was performed as described above. The efficacy of G595 was verified by Western blotting using the corresponding antibodies.

© 2023 The Authors

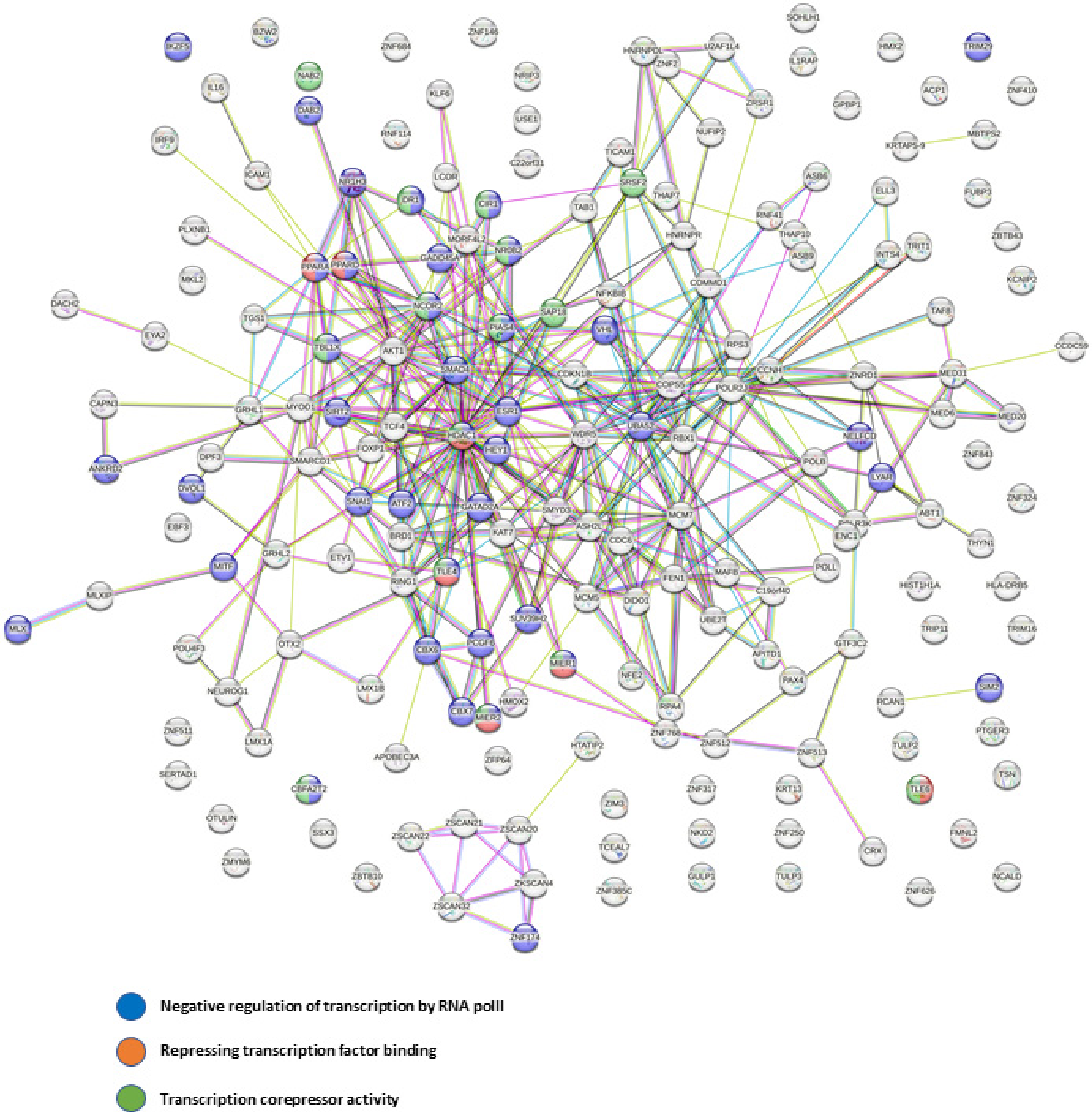

Figure EV5. Analysis of protein interactions for ERα partners identified by our screen.

Proteins whose interactions with ERα were induced by Tam and regulated by PRMT5 activity were analyzed using the STRING database. Proteins involved in the repression of transcription are highlighted in color.

 © 2023 The Authors
