# Supplementary material for: Nuclear PRMT5 is a biomarker of sensitivity to tamoxifen in ERα + breast cancer
Source: EMBO Mol Med. 2023 Jul 17;15(8):e17248. doi: 10.15252/emmm.202217248 (PMC10405064; doi:10.15252/emmm.202217248)
Supplement: Supplementary file 12 [file EMMM-15-e17248-s005.pdf]

EMBO Molecular Medicine

Nuclear PRMT5 is a biomarker of sensitivity to tamoxifen in ERα+ breast cancer

Coralie Poulard, Thuy Ha Pham, Youenn Drouet, Julien Jacquemetton, Ausra Surmielova, Benoite Mery, Lohay Kassem, Jonathan Reboulet, Christine Lasset, Isabelle Treilleux, Elisabetta Marangoni, Olivier Tredan, and Muriel Le Romancer
DOI: 10.15252/emmm.202217248

Corresponding author: Muriel Le Romancer (muriel.leromancer@lyon.unicancer.fr)

Review Timeline:

Submission Date:	13th Feb 23
Editorial Decision:	7th Mar 23
Revision Received:	22nd May 23
Editorial Decision:	16th Jun 23
Revision Received:	23rd Jun 23
Accepted:	26th Jun 23

Editor: Lise Roth

Transaction Report:

(Note: With the exception of the correction of typographical or spelling errors that could be a source of ambiguity, letters and reports are not edited. Depending on transfer agreements, referee reports obtained elsewhere may or may not be included in this compilation. Referee reports are anonymous unless the Referee chooses to sign their reports.)

7th Mar 2023

Dear Dr. Le Romancer,

Thank you for the submission of your manuscript to EMBO Molecular Medicine. We have now received feedback from the three reviewers who agreed to evaluate your manuscript. As you will see from the reports below, the referees acknowledge the interest of the study and are overall supporting publication of your work pending appropriate revisions.

Following further discussion with the referees, we agreed that identification of the exact residue targeted by PRMT5 would not be required, however an appropriate discussion and more information as to why you could not identify the methylated residue will be necessary.
Similarly, targeting of PRMT5 to sensitize breast cancer cells to tamoxifen could be done in vitro (in vivo experiments would be welcome but not requested).

Addressing all the other reviewers' concerns in full will be necessary for further considering the manuscript in our journal, and acceptance of the manuscript will entail a second round of review.

EMBO Molecular Medicine encourages a single round of revision only and therefore, acceptance or rejection of the manuscript will depend on the completeness of your responses included in the next, final version of the manuscript. For this reason, and to save you from any frustrations in the end, I would strongly advise against returning an incomplete revision.

If you would like to discuss further the points raised by the referees, I am available to do so via email or video. Let me know if you are interested in this option.

Revised manuscripts should be submitted within three months of a request for revision; they will otherwise be treated as new submissions, except under exceptional circumstances in which a short extension is obtained from the editor.

When submitting your revised manuscript, please carefully review the instructions that follow below. We perform an initial quality control of all revised manuscripts before re-review; failure to include requested items will delay the evaluation of your revision.

We require:

1) A .docx formatted version of the manuscript text (including legends for main figures, EV figures and tables). Please make sure that the changes are highlighted to be clearly visible.

2) Individual production quality figure files as .eps, .tif, .jpg (one file per figure). For guidance, download the 'Figure Guide PDF' (https://www.embopress.org/page/journal/17574684/authorguide#figureformat).

3) At EMBO Press we ask authors to provide source data for the main figures. Our source data coordinator will contact you to discuss which figure panels we would need source data for and will also provide you with helpful tips on how to upload and organize the files.

4) A .docx formatted letter INCLUDING the reviewers' reports and your detailed point-by-point responses to their comments. As part of the EMBO Press transparent editorial process, the point-by-point response is part of the Review Process File (RPF), which will be published alongside your paper.

5) A complete author checklist, which you can download from our author guidelines (https://www.embopress.org/page/journal/17574684/authorguide#submissionofrevisions). Please insert information in the checklist that is also reflected in the manuscript. The completed author checklist will also be part of the RPF.

6) It is mandatory to include a 'Data Availability' section after the Materials and Methods. Before submitting your revision, primary datasets produced in this study need to be deposited in an appropriate public database, and the accession numbers and database listed under 'Data Availability'. Please remember to provide a reviewer password if the datasets are not yet public (see https://www.embopress.org/page/journal/17574684/authorguide#dataavailability).

In case you have no data that requires deposition in a public database, please state so in this section "This study includes no data deposited in external repositories". Note that the Data Availability Section is restricted to new primary data that are part of this study.

7) For data quantification: please specify the name of the statistical test used to generate error bars and P values, the number (n) of independent experiments (specify technical or biological replicates) underlying each data point and the test used to calculate p-values in each figure legend. The figure legends should contain a basic description of n, P and the test applied.

Graphs must include a description of the bars and the error bars (s.d., s.e.m.). Please provide exact p values.

8) Our journal encourages inclusion of *data citations in the reference list* to directly cite datasets that were re-used and obtained from public databases. Data citations in the article text are distinct from normal bibliographical citations and should directly link to the database records from which the data can be accessed. In the main text, data citations are formatted as follows: "Data ref: Smith et al, 2001" or "Data ref: NCBI Sequence Read Archive PRJNA342805, 2017". In the Reference list, data citations must be labeled with "[DATASET]". A data reference must provide the database name, accession number/identifiers and a resolvable link to the landing page from which the data can be accessed at the end of the reference. Further instructions are available at .

9) We replaced Supplementary Information with Expanded View (EV) Figures and Tables that are collapsible/expandable online. A maximum of 5 EV Figures can be typeset. EV Figures should be cited as 'Figure EV1, Figure EV2" etc... in the text and their respective legends should be included in the main text after the legends of regular figures.
- For the figures that you do NOT wish to display as Expanded View figures, they should be bundled together with their legends in a single PDF file called *Appendix*, which should start with a short Table of Content. Appendix figures should be referred to in the main text as: "Appendix Figure S1, Appendix Figure S2" etc.
- Additional Tables/Datasets should be labeled and referred to as Table EV1, Dataset EV1, etc. Legends have to be provided in a separate tab in case of .xls files. Alternatively, the legend can be supplied as a separate text file (README) and zipped together with the Table/Dataset file.
See detailed instructions here:

10) For more information: There is space at the end of each article to list relevant web links for further consultation by our readers. Could you identify some relevant ones and provide such information as well? Some examples are patient associations, relevant databases, OMIM/proteins/genes links, author's websites, etc...

11) Author contributions: CRediT has replaced the traditional author contributions section because it offers a systematic machine readable author contributions format that allows for more effective research assessment. Please remove the Authors Contributions from the manuscript and use the free text boxes beneath each contributing author's name in our system to add specific details on the author's contribution. More information is available in our guide to authors.

12) Disclosure statement and competing interests: We updated our journal's competing interests policy in January 2022 and request authors to consider both actual and perceived competing interests. Please review the policy https://www.embopress.org/competing-interests and update your competing interests if necessary.

13) Every published paper now includes a 'Synopsis' to further enhance discoverability. Synopses are displayed on the journal webpage and are freely accessible to all readers. They include a short stand first (maximum of 300 characters, including space) as well as 2-5 one-sentences bullet points that summarizes the paper. Please write the bullet points to summarize the key NEW findings. They should be designed to be complementary to the abstract - i.e. not repeat the same text. We encourage inclusion of key acronyms and quantitative information (maximum of 30 words / bullet point). Please use the passive voice. Please attach these in a separate file or send them by email, we will incorporate them accordingly.

Please also suggest a striking image or visual abstract to illustrate your article as a PNG file 550 px wide x 300-600 px high.

14) As part of the EMBO Publications transparent editorial process initiative (see our Editorial at http://embomolmed.embopress.org/content/2/9/329), EMBO Molecular Medicine will publish online a Review Process File (RPF) to accompany accepted manuscripts.
In the event of acceptance, this file will be published in conjunction with your paper and will include the anonymous referee reports, your point-by-point response and all pertinent correspondence relating to the manuscript. Let us know whether you agree with the publication of the RPF and as here, if you want to remove or not any figures from it prior to publication.
Please note that the Authors checklist will be published at the end of the RPF.

EMBO Molecular Medicine has a "scooping protection" policy, whereby similar findings that are published by others during review or revision are not a criterion for rejection. Should you decide to submit a revised version, I do ask that you get in touch after three months if you have not completed it, to update us on the status.

I look forward to receiving your revised manuscript.

Yours sincerely,

Lise Roth

Lise Roth, PhD
Senior Editor
EMBO Molecular Medicine

EMBO Press participates in many Publish and Read agreements that allow authors to publish Open Access with reduced/no publication charges. Check your eligibility: https://authorservices.wiley.com/author-resources/Journal-Authors/open-access/affiliation-policies-payments/index.html

***** Reviewer's comments *****

Referee #1 (Comments on Novelty/Model System for Author):

Resistance to Tamoxifen (Tam) is a problem of major importance to counteract ERa-positive breast cancer evolution. In this regard, aberrant ERa post transcriptional modifications is known to contribute to inappropriate genes expressions underlying the emergence of this unfortunate resistance. Present manuscript concerns this topic : reported investigations concern ERa methylation by the methyl-transferase PRTM5. This factor controls the recruitment of co-repressors (SMRT, HDC1...) to the receptor upon Tam treatment so that it may achieve its therapeutic effect. Translocation of the ERa-PRMT5-COREPRESSOR machinery from the cytoplasm to the nucleus under Tam exposure occurs indeed solely in tumor cells responding to the curative effect of the antiestrogen. Hence, identification of a nuclear PRMT5 staining rate in breast cancer under Tam administration might become a new prognostic factor, if confirmed in subsequent clinical investigations. Moreover, search to increase this staining as well as evaluate its value for other SERMs may offer new therapeutic options.
This important information derives from a fundamental investigation performed with experimental models (breast cancer cell lines , patient-derived xenograft models) as well as from a complementary investigation clinical implying a significant group of patients , both studies being extremely well conducted generating a multitude of data that do not suffer of criticisms [Fig 5C ERa/SDMA (red dots; left) : sole not evident information of which pertinence is nevertheless established by a statistical analysis of 3 independent experiments].
Analysis of ERa interactome in cells treated or not with Tam in the presence of a specific PRTM5 inhibitor highlights the role of this antiestrogen in the repressive potency of PRTM5 on this interactome . Fig 8S is devoted to identify proteins involved in this repression (color). Could such proteins be more easily identified by citing them in the footnote? An increase of the intensity of the lines related to the relationships between these proteins might also be fruitfull .
Implication of deacetylases HDAC1 , SIRT2 and SAP18 in the repressive action of PRTM5 suggests a cooperative effect between all these factors at the level of ERa , complementary to histones (Fig 8) . This proposition may moreover explain the paradoxical action of PRTM5 at the level of the DBD (aa 176-251) which is devoid of NLS, as explained here under.
Taking into account that ERa accumulated in cell nucleus under Tam treatment is not implicated in growth inhibition (Legros N ,Cancer Chemother Pharmacol 39:38382, 1997), one may consider that its inhibitory effect (related to PRTM5 nuclear localisation ; present study) might be relevant to to a complex NLS-containing bypass system able to paliate the absence of a specific PRMT5 NLS. The aa 295-311 hub of the receptor implicated in its intracellular trafficking (and turnover) though its ability to recruit a panel of coregulators with post transcriptional potency may play this paliative role through its own 299-303 NLS motif (K303R mutation confers indeed estrogen hypersensitivity with associated low curative response not solely to aromatase inhibitors since Tam action is also affected ; S Fuqua). Implication of importins, evoked in the discussion of the manuscript, may contribute to this bipolar cooperative regulatory process. Importin a favors indeed
the shipment of ERa to the nucleus through a dual interactions between a NLS located at the interface of the DBD (PRMT5 target) and the hinge region (aa 266-269) with the 299-303 NLS of the aa295-311 hub (Kalyvianaki K Frontiers in Endocrinology 13:954629, 2022). Of course, this cooperative view is still speculative but if the authors find it suitable within the discussion , I invite them to incude it!

Remark of minor importance :p17 L8 ...SMRT was not for TFF1 and XBP1

Referee #1 (Remarks for Author):

Excellent work!

Referee #2 (Remarks for Author):

Poulard et al. reported that the nuclear abundance of PRMT5 is the predictive biomarker for the sensitivity of ER-positive breast cancer cells to tamoxifen. They show that PRMT5 methylates ERα upon the tamoxifen treatment and promotes the interaction of ERα with its co-repressors. The study concludes that the nuclear expression of PRMT5 might be exploited as a predictive marker to decide on tamoxifen treatment. Overall, the paper reports interesting findings which have clinical relevance. The experiments are well-designed, and the results are convincing, which support the conclusions.
However, the authors should address the following concerns.
1. Though the findings suggest that the treatment with tamoxifen increases the PRMT5-mediated methylation of ERα, it is not clear whether the tamoxifen treatment promotes the nuclear localization of PRMT5 or not. This can be sorted out by treating the MCF7 cells with tamoxifen and investigating the sub-cellular distribution of PRMT5.
2. The in vitro methylation experiments revealed that PRMT5 methylates the DNA binding domain of ERα, but methylation of full-length ERα by PRMT5 was not observed. Please comment on this.
3. The ChIP data (Fig. 4B) indicated the enhanced recruitment of ERα and SMRT to the promoters of ERα target genes upon treatment with tamoxifen. It is unclear whether this recruitment leads to the suppression of the expression of these genes. The authors need to clarify this through experiments for a better mechanistic understanding of the tamoxifen- PRMT5 axis.
4. In page 15, paragraph 2, line 3, the authors stated that "The images obtained revealed specific red dots in the nucleus of MCF7 cell". But I could see the red spots outside the nuclei as well. This sentence may be rephrased as "The images obtained revealed specific red dots predominantly in the nucleus of MCF7 cell".
5. In page 14, paragraphs 2 and 3, lines 3 and 7, the authors stated the "PRMT5 nuclear expression" as "PRMT5 expression" and that needs to be corrected.
6. Most western blots throughout the manuscript lack the indication of molecular weight markers.

Referee #3 (Comments on Novelty/Model System for Author):

Poulard et al found elevated nuclear PRMT5 arginine methyltransferase as a predictive marker for sensitivity to tamoxifen and, ultimately, breast cancer patient survival. Mechanistically, they found that tamoxifen treatment correlates with the presence of PRMT5 in the nucleus of breast cancer cells, where it methylates the estrogen receptor ERalpha. This in turn is associated with increased interaction of ERalpha with transcriptional co-repressors and the recruitment of these repressive complexes to ERalpha-responsive promoters.

Overall technical quality is high, but the lack of rescue experiments and functional readouts render the findings somewhat correlative in nature.

Statistical analysis is state-of-the-art (with e.g. the use of a discovery and validation cohort to truly assess the predictive potential of nuclear PRMT5 levels).

Findings are clinically relevant in that they uncover a novel biomarker for sensitivity to tamoxifen - a current treatment regimen for breast cancer - and identify a potential novel therapeutic avenue in targeting PRMT5 to the nucleus in resistant breast tumours.

Referee #3 (Remarks for Author):

Poulard et al found elevated nuclear PRMT5 arginine methyltransferase as a predictive marker for sensitivity to tamoxifen and, ultimately, breast cancer patient survival. Mechanistically, they found that tamoxifen treatment correlates with the presence of PRMT5 in the nucleus of breast cancer cells, where it methylates the estrogen receptor ERalpha. This in turn is associated with increased interaction of ERalpha with transcriptional co-repressors and the recruitment of these repressive complexes to ERalpha-responsive promoters.

Overall, this study provides new insight into a highly clinically-relevant problem and uncovers a potentially novel therapeutic avenue.

While generally technically sound, there are a few lines of experimentations that are lacking to allow for stronger conclusions to be made. There are also a few recommended adjustments/additions to the currently included experiments that would reassure about the validity of results interpretation.

Specific Points:

Fig. 2E: G595 decreases the ERa-PRMT5 interaction. This is interpreted as meaning that the enzymatic activity of PRMT5 is required to stabilize the interaction. This reasoning could be expanded to also suggest that in the presence of inhibitors, PRMT5 sub cellular localization may be affected. Such an observation has been made for PRMT1 by the Fackelmayer group.

Fig. 3: while the authors mention in the Discussion that they have failed at identifying the residue targeted by PRMT5 on nuclear

ERa, it would warrant further clarification. At the very least, it would be important to perform an in vivo methylation assay in the presence or absence of G595 or PRMT5 siRNAs (and look at nuclear ERa being 3H-labeled). These experiments could also be performed using mass spec, where it should be feasible to assess ERa methylation status (and also potentially map the modified residue if the proper protease(s) is(are) used for the analysis).

Fig. 3D: It would be more appropriate to perform the reciprocal experiment here - i.e. IP ERa (maybe from a nuclear fraction) and then blot with the sDMA antibody. As perform, one cannot eliminate the possibility that the relative abundance of ERa being pulled down is through an indirect interaction via one or more other methylated proteins recognized by the sDMA antibody. If the experiment as presented is kept, the dilution/amount of sDMA antibody used for IPs is not indicated in Supp. Table 2.

Figure 4B, does increased association of SMRT with promoters lead to gene repression? Could do RT-qPCR. Also, Is the Rme-dependent interaction between ERalpha and SMRT direct? Which domain of SMRT is involved? Could at least discuss from the literature whether ERalpha and SMRT interact directly, if not could any of the factors identified in S8 be involved in an interaction with Rme modified ERalpha, through a TUDOR domain for example? Figure S8 is not mentioned in the manuscript. I think it is referred as Data EV1 in the result section.

Where relevant and feasible, the manuscript would be strengthened by the inclusion of rescue experiments (e.g. overexpressing on top of the inhibitor or expression an siRNA-resistant allele).

Finally, the inclusion of at least one proof-of-concept experiment showing that targeting PRMT5 to the nucleus and/or increasing nuclear meR-ERa is able to sensitize breast cancer cells to tamoxifen would greatly strengthen the clinical relevance of the authors observations.

Responses to reviewers.

Referee#1 :(Comments on Novelty/Model System for Author):

Resistance to Tamoxifen (Tam) is a problem of major importance to counteract ERa-positive breast cancer evolution. In this regard, aberrant ERa post transcriptional modifications is known to contribute to inappropriate genes expressions underlying the emergence of this unfortunate resistance. Present manuscript concerns this topic : reported investigations concern ERa methylation by the methyl-transferase PRTM5. This factor controls the recruitment of co-repressors (SMRT, HDC1...) to the receptor upon Tam treatment so that it may achieve its therapeutic effect. Translocation of the ERa-PRMT5-COREPRESSOR machinery from the cytoplasm to the nucleus under Tam exposure occurs indeed solely in tumor cells responding to the curative effect of the antiestrogen. Hence, identification of a nuclear PRMT5 staining rate in breast cancer under Tam administration might become a new prognostic factor, if confirmed in subsequent clinical investigations. Moreover, search to increase this staining as well as evaluate its value for other SERMs may offer new therapeutic options.
This important information derives from a fundamental investigation performed with experimental models (breast cancer cell lines , patient-derived xenograft models) as well as from a complementary investigation clinical implying a significant group of patients , both studies being extremely well conducted generating a multitude of data that do not suffer of criticisms [Fig 5C ERa/SDMA (red dots; left) : sole not evident information of which pertinence is nevertheless established by a statistical analysis of 3 independent experiments].

Analysis of ERa interactome in cells treated or not with Tam in the presence of a specific PRTM5 inhibitor highlights the role of this antiestrogen in the repressive potency of PRTM5 on this interactome . Fig 8S is devoted to identify proteins involved in this repression (color). Could such proteins be more easily identified by citing them in the footnote? An increase of the intensity of the lines related to the relationships between these proteins might also be fruitfull.

Response :

We thank the reviewer for his/her interest in our work. Concerning his/her request, the modification has been done.

-Implication of deacetylases HDAC1 , SIRT2 and SAP18 in the repressive action of PRTM5 suggests a cooperative effect between all these factors at the level of ERa , complementary to histones (Fig 8) .

This proposition may moreover explain the paradoxical action of PRTM5 at the level of the DBD (aa 176-251) which is devoid of NLS, as explained here under. Taking into account that ERa accumulated in cell nucleus under Tam treatment is not implicated in growth inhibition (Legros N ,Cancer Chemother Pharmacol 39:38382, 1997), one may consider that its inhibitory effect (related to PRTM5 nuclear localisation ; present study) might be relevant to to a complex NLS-containing bypass system able to paliate the absence of a specific PRMT5 NLS. The aa 295-311 hub of the receptor implicated in its intracellular trafficking (and turnover) though its ability to recruit a panel of coregulators with post transcriptional potency may play this paliative role through its own 299-303 NLS motif (K303R mutation confers indeed estrogen hypersensitivity with associated low curative response not solely to aromatase inhibitors since Tam action is also affected ; S Fuqua). Implication of importins, evoked in

the discussion of the manuscript, may contribute to this bipolar cooperative regulatory process. Importin a favors indeed the shipment of ERa to the nucleus through a dual interactions between a NLS located at the interface of the DBD (PRMT5 target) and the hinge region (aa 266-269) with the 299-303 NLS of the aa295-311 hub (Kalyvianaki K Frontiers in Endocrinology 13:954629, 2022). Of course, this cooperative view is still speculative but if the authors find it suitable within the discussion , I invite them to incude it!

<u>Response :</u>

We agree with the reviewer that importins are also involved in ERα transportation in the nucleus and that it could be a way to import PRMT5 into the nucleus. However, we think that upon Tam treatment, ERα is unlikely involved in PRMT5 translocation in the nucleus in MCF7 tumors. Indeed, ERα is already present in the nucleus without Tam treatment, suggesting that other actors are involved. This is why it will be of interest in the future to identify which proteins are implicated in PRMT5 binding to importins to enter within the nucleus and also how its export from the nucleus is regulated.

-**Remark of minor importance :p17 L8 ...SMRT was not for TFF1 and XBP1.**

<u>Response :</u>
We have modified the sentence as requested.

Referee #2 (Remarks for Author):

Poulard et al. reported that the nuclear abundance of PRMT5 is the predictive biomarker for the sensitivity of ER-positive breast cancer cells to tamoxifen. They show that PRMT5 methylates ERα upon the tamoxifen treatment and promotes the interaction of ERα with its co-repressors. The study concludes that the nuclear expression of PRMT5 might be exploited as a predictive marker to decide on tamoxifen treatment. Overall, the paper reports interesting findings which have clinical relevance. The experiments are well-designed, and the results are convincing, which support the conclusions. However, the authors should address the following concerns.

1. Though the findings suggest that the treatment with tamoxifen increases the PRMT5-mediated methylation of ERα, it is not clear whether the tamoxifen treatment promotes the nuclear localization of PRMT5 or not. This can be sorted out by treating the MCF7 cells with tamoxifen and investigating the sub-cellular distribution of PRMT5.

Response :
We appreciate the interest shown by Reviewer #2 for our findings. We have addressed this point in Figure 6 A where we showed that upon Tam treatment, PRMT5 translocates into the nucleus of tumoral cells in fixed tumors from MCF7 engrafted cells in mice. To make these results clearer, we have quantified PRMT5 expression in the nucleus with the same H-score used for the human tumors (Figure 1A and 1B) as described in the methods section. We can see from the results calculated with 7 control mice and 7 Tam-treated mice that the average nuclear PRMT5 H-score is 8 for control tumors and 150 for Tam-treated tumors, highlighting that Tam has a major effect on PRMT5 nuclear translocation.
As requested, we performed the experiment in MCF7 cells treated by Tam and conducted cell fractionation to evaluate PRMT5 expression in the cytoplasmic and nuclear fractions. Figure A showed the same result but to a lesser extend. Obviously, *in vitro*, it is difficult to maintain PRMT5 in the

nucleus. On the contrary, in mouse xenografts, we observed a strong expression of PRMT5 in the nucleus, suggesting an active import associated with an inhibition of export that could be regulated by external stimuli from the microenvironment.

As these results in MCF7 cells *in vitro* do not provide striking information in the article, we suggest not to add them in the manuscript.

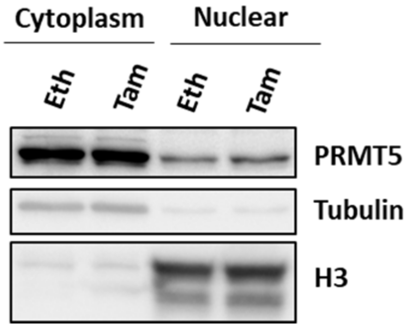

Figure A: Study of PRMT5 nuclear translocation upon Tam treatment. *MCF7 cells were fractionated and each fraction was assessed for PRMT5n tubulin and Histone H3 expression by Western blotting.*

2. The in vitro methylation experiments revealed that PRMT5 methylates the DNA binding domain of ERα, but methylation of full-length ERα by PRMT5 was not observed. Please comment on this.

Response :

We did not perform experiments on the full length ERα because the protein is difficult to express. In addition, from our previous experience with PRMT1, although ERα DNA binding domain was highly methylated by PRMT1 (Le Romancer et al, Mol Cell, 2008) we were unable to measure any methylation on the full length protein, likely because a lack of the right conformation and access to the modified arginine residue. For this reason, we preferred to address the methylation of the full-length ERα in cells in endogenous conditions by two complementary techniques : PLA and IP (Figure 3C, D,E).

3. The ChIP data (Fig. 4B) indicated the enhanced recruitment of ERα and SMRT to the promoters of ERα target genes upon treatment with tamoxifen. It is unclear whether this recruitment leads to the suppression of the expression of these genes. The authors need to clarify this through experiments for a better mechanistic understanding of the tamoxifen- PRMT5 axis.

Response :

We thank the reviewer for this relevant comment. We have now performed RT-QPCR to evaluate the expression of the 3 ERα target genes: TFF1, GREB1 and XBP1 upon Tam treatment and found that their expression is significantly decreased. These results have been added as Figure 4C.

4. In page 15, paragraph 2, line 3, the authors stated that "The images obtained revealed specific red dots in the nucleus of MCF7 cell". But I could see the red spots outside the nuclei as well. This sentence may be rephrased as "The images obtained revealed specific red dots predominantly in the nucleus of MCF7 cell".

Response :

The sentence has been corrected as requested.

5. In page 14, paragraphs 2 and 3, lines 3 and 7, the authors stated the "PRMT5 nuclear expression" as "PRMT5 expression" and that needs to be corrected.

Response :

When we write PRMT5 nuclear expression, it is to highlight that it is only the expression of PRMT5 in the nucleus that is taken into account.

6. Most western blots throughout the manuscript lack the indication of molecular weight markers.

Response :

We have added the missing molecular weight markers for the following figures as requested: Figure 2B, 2C, 2E, Figure 3C, 3D, 3E, Figure 4A, Figure 5B, Appendix S2 and S3, Figure EV2D, Figure EV3, Figure EV4.

Referee #3 (Remarks for Author):

Poulard et al found elevated nuclear PRMT5 arginine methyltransferase as a predictive marker for sensitivity to tamoxifen and, ultimately, breast cancer patient survival. Mechanistically, they found that tamoxifen treatment correlates with the presence of PRMT5 in the nucleus of breast cancer cells, where it methylates the estrogen receptor ERalpha. This in turn is associated with increased interaction of ERalpha with transcriptional co-repressors and the recruitment of these repressive complexes to ERalpha-responsive promoters.

Overall, this study provides new insight into a highly clinically-relevant problem and uncovers a potentially novel therapeutic avenue.

While generally technically sound, there are a few lines of experimentations that are lacking to allow for stronger conclusions to be made. There are also a few recommended adjustments/additions to the currently included experiments that would reassure about the validity of results interpretation.

Specific Points:

Fig. 2E: G595 decreases the ERa-PRMT5 interaction. This is interpreted as meaning that the enzymatic activity of PRMT5 is required to stabilize the interaction. This reasoning could be expanded to also suggest that in the presence of inhibitors, PRMT5 sub cellular localization may be affected. Such an observation has been made for PRMT1 by the Fackelmayer group.

Response :

The reviewer is right, we have now assessed the impact of G595 on PRMT5 localization. For this purpose, we performed 2 experiments: one in paraffin-embedded sections from a fresh breast tumor incubated with and without G595 and observed that upon G595 treatment PRMT5 was excluded from the nucleus. This result has been added as Figure 2F. We also performed the same experiment in MCF7 cells and after cell fractionation coupled to Western blots, we observed the same effect. This result has been added as Figure S2B. In conclusion, G595 decreases ERα/PRMT5 interaction by translocating PRMT5 from the nucleus to the cytoplasm.

Fig. 3: while the authors mention in the Discussion that they have failed at identifying the residue targeted by PRMT5 on nuclear ERa, it would warrant further clarification. At the very least, it would be important to perform an in vivo methylation assay in the presence or absence of G595 or PRMT5 siRNAs (and look at nuclear ERa being 3H-labeled). These experiments could also be performed using mass spec, where it should be feasible to assess ERa methylation status (and also potentially map the modified residue if the proper protease(s) is(are) used for the analysis).

<u>Response :</u>

We have tried several approaches to identify the methylated residue in ERα but failed because of the sequence/structure of the DBD. In the figure B, we can see that the DBD contains 6 arginine residues (highlighted in red). This domain is very structured as it forms 2 zing finger domains (lower panel). We first tried to identify the methylated arginine residue by site-directed mutagenesis coupled to methylation in vitro experiments. Unfortunately, probably because of the structured domain, some mutants could not be produced in bacteria, and several point mutations were decreasing partially the level of ERα methylation, preventing the identification of the methylated arginine residue. Next, we decided to immunoprecipitate ERα from MCF7 cells treated with Tam and identify the methylated residue by mass spectrometry experiments. Once again we faced problems, as the DBD sequence was never covered, probably because of the presence of basic residues that are cut by the trypsin digestion making small non visible peptides for mass spec analysis (K highlighted in green). In the literature, another group described a similar failure for measuring the methylation by SMYD2 on the K266 of ERα located in the same region and talk about the same concerns (*Zhang et al, PNAS, 2013, Regulation of estrogen receptor a by histone methyltransferase SMYD2-mediated protein methylation*).

This point has been added in the discussion section as requested.

^{176}ESAKETRYCAVCNDYASGYHYGVWSCEGCKAFFKRSIQGHNDYMCPATNQCTIDKNRRKSCQACRLRKCYEVGMM^{251}

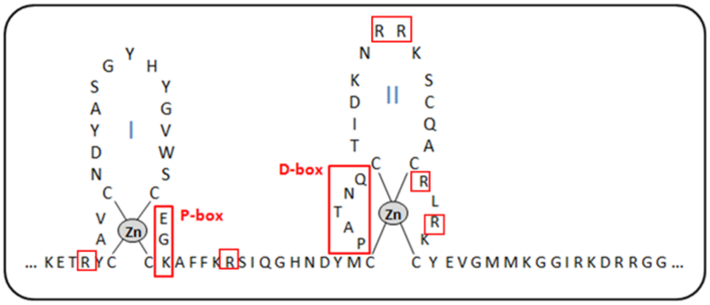

Figure B : *Sequence of structure of ERα DBD with arginine residues highlighted*

Fig. 3D: It would be more appropriate to perform the reciprocal experiment here - i.e. IP ERa (maybe from a nuclear fraction) and then blot with the sDMA antibody. As perform, one cannot eliminate the possibility that the relative abundance of ERa being pulled down is through an indirect interaction via one or more other methylated proteins recognized by the sDMA antibody. If the experiment as presented is kept, the dilution/amount of sDMA antibody used for IPs is not indicated in Supp. Table 2.

<u>Response :</u>

As suggested by the reviewer, we have performed the reciprocal experiment but unfortunately, we did not see any band corresponding to methylated ERα (Figure C). This is not surprising as protein methylation is often affected in a small fraction of the protein. This is the reason why we chose to perform PLA which amplifies the signal as well as IP with SDMA antibody to enrich for methylated proteins. In addition, as we observed ERα methylation in vitro we have no doubt that the receptor is methylated.

Moreover, steroid receptors seem to be privileged targets for PRMT5 as we found that GR and AR are also methylated by PRMT5 (Poulard et al, Methods 2019, Alegre-Martí A, Science advances, 2023).

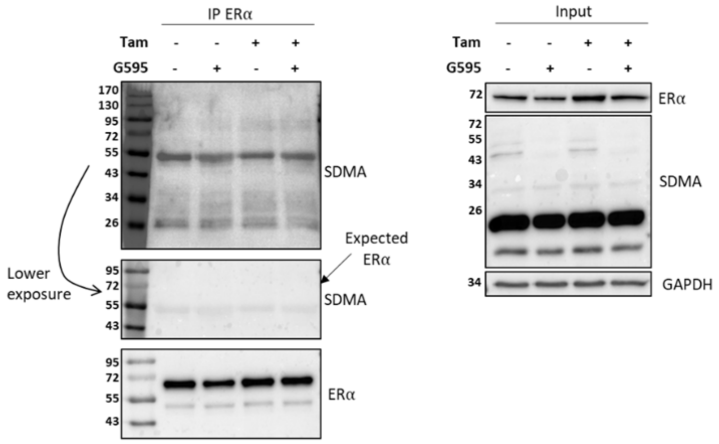

Figure C : Study of ERα methylation SDMA by immunoprecipitating ERα.

MCF7 cells were treated with or without Tam in the presence or absence of PRMT5 inhibitor, protein lysates were then immunoprecipitated with an anti-ERα antibody and revealed with an anti-ERα antibody or the pan methyl antibody.

In addition, to validate in vivo that ERα methylation was performed by PRMT5, from the same embedded fresh breast tumor treated with G595 discussed earlier, we measured ERα methylation SDMA by PLA and observed that the signal disappeared totally after G595 treatment (see Figure S9B).

Figure 4B, does increased association of SMRT with promoters lead to gene repression? Could do RT-qPCR. Also, Is the Rme-dependent interaction between ERalpha and SMRT direct? Which domain of SMRT is involved? Could at least discuss from the literature whether ERalpha and SMRT interact directly, if not could any of the factors identified in S8 be involved in an interaction with Rme modified ERalpha, through a TUDOR domain for example?

<u>Response :</u>

As also requested by reviewer 2, we performed qPCR experiments and found that the 3 genes are down regulated by tamoxifen. The results are presented in Figure 4C.

For the mechanism of ERα/SMRT interaction, it is true that it is an important point that should be more emphasized in the discussion section. From the literature, we know that SMRT binding to ERα is direct (*VARLAKHANOVA ET AL, Mol Cell Biol, 2010*). In this paper, the authors dissected the domains of ERα and SMRT involved in their interaction. It appears that SMRT binds ERα DBD on two regions: one in the

N terminus part (1-289aa) and the other in the C-terminus part (1210-2470 aa). In vitro, this interaction is insensitive to tam treatment. As SMRT does not possess a Tudor domain recognizing methylarginine, we can speculate that in vivo, ERα methylation by Tam treatment could increase its interaction with SMRT by modifying the conformation facilitating the interaction or also by inhibiting the binding of a protein bound to ERα that blocks the binding of SMRT.

Figure S8 is not mentioned in the manuscript. I think it is referred as Data EV1 in the result section.

Response :

We verified and Figure S8 was cited in page 17 of the manuscript. Data EV1 contains the table of the proteins that differentially bind ERα upon G595 treatment.

Where relevant and feasible, the manuscript would be strengthened by the inclusion of rescue experiments (e.g. overexpressing on top of the inhibitor or expression an siRNA-resistant allele).

Response :

To answer to the reviewer's concern, as we used pools containing 4 different siRNAS targeting PRMT5 it would be difficult to overexpress an siRNA-resistant plasmid. We chose to overexpress PRMT5 with its cofactor MEP50 on the top of the inhibitor. We first tried to validate that overexpressing PRMT5/MEP50 rescues the inhibition of SDMA. Unfortunately, as you can see in the Figure D, PRMT5 expression did not increase the global level of SDMA nor revert the effect of G595. However, it is unlikely that ERα methylation is not performed by PRMT5 as both proteins interact and ERα methylation is decreased with both the G595 and a pool of siRNAs targeting specifically PRMT5. In addition, in vivo, PRMT5 nuclear localization is associated with ERα SDMA methylation (Figure 6B) and an experiment using an ex vivo organotypic culture assay, in which the tumor microenvironment is closely mimicked, we found that ERα/SDMA is strongly decreased upon G595 treatment (added as a new result in Figure S4B).

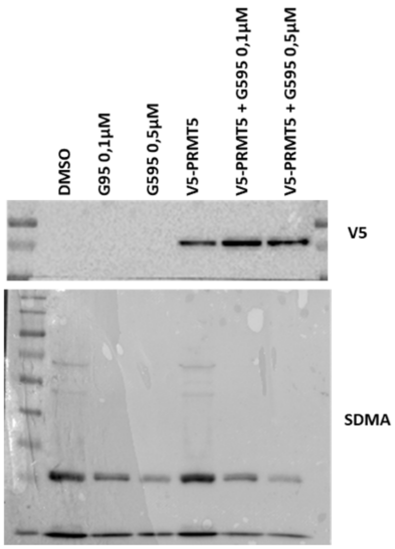

Figure D : Study of SMDA methylation upon PRMT5 overexpression

MCF7 cells were transfected with a plasmid expressing V5-PRMT5 in the presence or in absence of G595. Then, V5-PRMT5 expression was validated and SMDA level was assessed by Western blotting.

Finally, the inclusion of at least one proof-of-concept experiment showing that targeting PRMT5 to the nucleus and/or increasing nuclear meR-ERa is able to sensitize breast cancer cells to tamoxifen would greatly strengthen the clinical relevance of the authors observations.

Response :

We agree with the reviewer that this is a crucial point to force PRMT5 to stay in the nucleus where it exerts its anti-tumoral activity. However, as mentioned in the discussion, very little information is available about the regulation of PRMT5 shuttling. It is the topic of the study we are currently investigating to identify partners of PRMT5 involved in the regulation of its import in the nucleus as well of its export from the nucleus. Concerning the idea of increasing met ERα-SDMA, we thought that inhibiting the demethylase would be a good way. So far, JMJD6 is the only known arginine demethylase and we have published several years ago that JMJD6 regulates ERα methylation by PRMT1 (ADMA). On the contrary, we found that JMJD6 is not involved in the regulation of ERα methylation SDMA (data not shown).

16th Jun 2023

Dear Muriel,

Thank you for the submission of your revised manuscript to EMBO Molecular Medicine. We have now received the reports from referees #2 and #3 who re-reviewed your manuscript. As you will see, both referees are supportive of publication pending minor revisions, and we will therefore be able to accept your manuscript once the following points will be addressed:

1/ Referees' comments: please address the remaining concerns/questions from the referees in writing.

2/ Main manuscript text:
- Please remove the yellow highlights, accept all previous changes, and only keep in track changes mode any new modification.
- Materials and methods:
o Cell culture: please indicate whether the cells were authenticated and tested for mycoplasma contamination and update the checklist accordingly.
o Human samples: please add a statement that the experiments conformed to the principles set out in the WMA Declaration of Helsinki and the Department of Health and Human Services Belmont Report.
o Please provide the origin and concentration used for all reagents.
o Statistics: please include a statement about randomization, blinding and inclusion/exclusion criteria, and update the checklist accordingly.
- Please add a heading "Data Availability" after the Materials and Methods (above "This study includes no data deposited in external repositories").
- Please merge the "Funding" with the "Acknowledgements" section
- Table 1 and Table 2 and their legends should be embedded in the manuscript (after main figure legends) and need to stay editable and black & white

3/ Figures and Appendix:
- Please provide exact p values, not a range, in all figures or in their legends.
- Please define the scale bars in the legends of the figures (some are not legible, e.g. Figure 2D)
- Please make sure all figures/figure panels are referenced in the text (callout for Appendix Table S5 is currently missing).
- Please remove the EV figures from the manuscript file.
- Please provide the Appendix without highlighting and track changes and in pdf format.
- Please insert the legend for Dataset EV1 in the same excel file in a separate tab.

4/ Source Data: Thank you for providing Source Data. Please upload them as 1 file per figure.

5/ The paper explained: I included minor edits in your text, please let me know if you agree with the following or amend as you see fit, and include it in the main manuscript file.
Problem
Tamoxifen is widely used as an anti-estrogen to treat ERα-positive breast cancers in pre-menopausal women. However, 25% of patients relapse due to resistance to treatment and there is currently no predictive biomarker for tamoxifen sensitivity. A better understanding of the mechanisms underlying resistance is essential to develop innovative strategies to improve clinical outcome for patients with endocrine-resistant cancers.

Results
Our study showed in two independent cohorts of breast tumors that a high nuclear expression of PRMT5 is associated with a prolonged survival of tamoxifen-treated patients. We also identified nuclear PRMT5 as a key factor in the effects of tamoxifen. Importantly, in vivo experiments revealed that tamoxifen triggers PRMT5 translocation to the nucleus of tumor cells where it methylates ERα. This event is a prerequisite for the recruitment of transcriptional corepressors to the promoters of target genes triggering repression of transcription and subsequent decrease in cell proliferation.

Impact
These results have potential clinical applications at diagnosis as they suggest that a high nuclear PRMT5 expression constitutes a predictive biomarker of tamoxifen sensitivity for pre-menopausal women. Future investigations should help determine a way to target PRMT5 to the nucleus and thus improve tamoxifen sensitivity.

6/ Synopsis: Thank you for providing a synopsis text and image. Please upload the image separately in jpeg, TIFF or png format and sized 550 pixels wide x 200-600 pixels high. Make sure that the text remains legible.

7/ As part of the EMBO Publications transparent editorial process initiative (see our Editorial at http://embomolmed.embopress.org/content/2/9/329), EMBO Molecular Medicine will publish online a Review Process File (RPF)

to accompany accepted manuscripts.
This file will be published in conjunction with your paper and will include the anonymous referee reports, your point-by-point response and all pertinent correspondence relating to the manuscript. Let us know whether you agree with the publication of the RPF and as here, if you want to remove or not any figures from it prior to publication.
Please note that the Authors checklist will be published at the end of the RPF.

I look forward to receiving your revised manuscript as soon as possible,

With kind regards,

Lise

Lise Roth, PhD
Senior Editor
EMBO Molecular Medicine

***** Reviewer's comments *****

Referee #2 (Remarks for Author):

The reviewers' comments were addressed adequately. The manuscript is now suitable for publication.

The authors may wish to incorporate the following minor corrections.

Page 12, last paragraph, line 3:
"The C fragment containing the DNA binding domain ..." may be rephrased as "The C-terminal fragment containing the DNA binding domain ..."

Page 14, second paragraph, line 3:
"chromatin immunoprecipitation (ChiP)" The abbreviation "ChiP" may be written in the usual form as "ChIP"

Referee #3 (Comments on Novelty/Model System for Author):

Poulard et al found elevated nuclear PRMT5 arginine methyltransferase as a predictive marker for sensitivity to tamoxifen and, ultimately, breast cancer patient survival. Mechanistically, they found that tamoxifen treatment correlates with the presence of PRMT5 in the nucleus of breast cancer cells, where it methylates the estrogen receptor ERalpha. This in turn is associated with increased interaction of ERalpha with transcriptional co-repressors and the recruitment of these repressive complexes to ERalpha-responsive promoters.

Overall technical quality is high, but the lack of rescue experiments and functional readouts render the findings somewhat correlative in nature. Rescue experiments were requested as part of my previous reviews, but unfortunately, could not be successfully performed - so this comment remains valid.

Statistical analysis is state-of-the-art (with e.g. the use of a discovery and validation cohort to truly assess the predictive potential of nuclear PRMT5 levels).

Findings are clinically relevant in that they uncover a novel biomarker for sensitivity to tamoxifen - a current treatment regimen for breast cancer - and identify a potential novel therapeutic avenue in targeting PRMT5 to the nucleus in resistant breast tumours.

Referee #3 (Remarks for Author):

We thank the Author for their attempt at addressing concerns identified in my previous reviews.

Most have now been satisfactorily addressed, either through changes within the text of the manuscript or through new

experiments.

Unfortunately, there are a few requested experiments that did not work.

For the nuclear localization in MCF7 following TAM treatment, did the author attempt to monitor localization through IF microscopy? Or performing the experiment with an exogenous GFP-PRMT5 maybe? (which would allow to look at the dynamics of nuclear translocation using FLIP e.g...).

For the methylation of nuclear ERa, the author did not mention whether they had tried, as suggested, a so-called 'in vivo methylation assay'? using 3H-Meth to label cells in the presence of translation inhibitors.. +/- te presence of PRMT5 siRNA or inhibitor.

Also, in the IP of ERa followed by blotting with sDMA-specific Ab - which sDMA-specific Ab was tried? SYM10? SYM11? Other? As these, despite what is advertised by vendors, all have some degree of preference for certain motifs around the actual methylated residue.

Finally, for the proof-of-concept experiment suggested, this reviewer agrees that it may be outside the scope of the current study to uncover the full mechanism of nuclear localization involved, but how about 'artificially' forcing PRMT5 to the nucleus with the addition of a strong NLS sequence?

Responses to reviewers.

Referee #2 (Remarks for Author):

The reviewers' comments were addressed adequately. The manuscript is now suitable for publication.

The authors may wish to incorporate the following minor corrections.

Page 12, last paragraph, line 3:
"The C fragment containing the DNA binding domain ..." may be rephrased as "The C-terminal fragment containing the DNA binding domain ..."

Page 14, second paragraph, line 3:
"chromatin immunoprecipitation (ChiP)" The abbreviation "ChiP" may be written in the usual form as "ChIP"

Response:

-We are happy to see that reviewer 2 was satisfied with our corrections.

We have not modified the sentence as it concerns the domain C of the receptor and it is not located in the C-terminal part of the protein.

-We have, however, followed his/her second recommendation and written “ChIP” in the revised manuscript.

Referee #3 (Remarks for Author): We thank the Author for their attempt at addressing concerns identified in my previous reviews.

Most have now been satisfactorily addressed, either through changes within the text of the manuscript or through new experiments.

We thank the reviewer for noting that we addressed the concerns that could be adequately performed or that had an impact on the general message of our manuscript.

Unfortunately, there are a few requested experiments that did not work.

For the nuclear localization in MCF7 following TAM treatment, did the author attempt to monitor localization through IF microscopy? Or performing the experiment with an exogenous GFP-PRMT5 maybe? (which would allow to look at the dynamics of nuclear translocation using FLIP e.g...).

Response :

We believe that through this concern, referee#3 is seeking further proof of the mechanisms of nuclear translocation following Tam treatment in a cellular model and to facilitate the study of this intricate shuttling mechanism. We attempted, as suggested, to perform IF experiments to observe PRMT5 nuclear translocation in MCF7 cells. However, the result was not striking. It is difficult to observe its translocation in cells compared to results we display in a more complex and in vivo model MCF7-engrafted mice (Figure 6A). We hypothesize that this discrepancy is probably due to a regulation of PRMT5 shuttling by signaling pathways regulated by the tumor microenvironment that are absent in cell culture. We suppose that in culture, PRMT5 enters the nucleus, methylates ERα (a stable mark) and then because of its three nuclear export signals, rapidly exits the nucleus. Our team is focusing on this shuttling mechanism and this will hopefully be the object of future manuscripts.

For the methylation of nuclear ERa, the author did not mention whether they had tried, as suggested, a so-called 'in vivo methylation assay'? using 3H-Meth to label cells in the presence of translation inhibitors.. +/- the presence of PRMT5 siRNA or inhibitor.

Response :

With this experiment, we believe the referee seeks confirmation of the methylation of the receptor. However, we did not try to perform such experiments in our lab (which is not accredited for such experiments). From the literature, we understand that this technique works for constitutive methylation, Here, it will be more tricky to perform as ERα is methylated mostly upon Tam treatment. Throughout the manuscript, we have demonstrated ERα methylation in vitro (Figure 3B), in cells (Figure 3C,D,E) and in tumors (Figure S4A). Moreover, we detected the signal for ERα methylation only in ERα-positive tumors. However, if the reviewer wants it, we can discuss this point in the discussion section.

Also, in the IP of ERa followed by blotting with sDMA-specific Ab - which sDMA-specific Ab was tried? SYM10? SYM11? Other? As these, despite what is advertised by vendors, all have some degree of preference for certain motifs around the actual methylated residue.

Response :

For all our experiments, we first tested the different antibodies recognizing SDMA, but only the SDMA antibody from Cell Signaling gave specific results (see Table S2). As mentioned by the referee, this is probably due to the preference of motifs around the target methylated residue.

Finally, for the proof-of-concept experiment suggested, this reviewer agrees that it may be outside the scope of the current study to uncover the full mechanism of nuclear localization involved, but how about 'artificially' forcing PRMT5 to the nucleus with the addition of a strong NLS sequence?

Response:

As explained in the first response to the referee, we are currently trying to elucidate the shuttling to the nucleus. We have previously performed experiments to force the translocation using NLS sequences, but unfortunately PRMT5 was still mainly localized in the cytoplasm, probably because of its 3 NES that favor its exit from the nucleus. Our hypothesis is that in mice xenografted with MCF7

cells, Tam induces a nuclear translocation, possibly via an as yet unidentified event (such as phosphorylation), which will impede the binding of PRMT5 to exportin, thus retaining the protein in the nucleus. Such experiments would mainly help us understand the exact mechanisms of shuttling, but our primary aim in the future is to force PRMT5 shuttling to the nucleus via natural means or drugs, by targeting key actors in endogenous cells that could be extended in vivo in mice and in patients to improve their response to Tam.

26th Jun 2023

Dear Muriel,

Thank for providing the revised files. I am pleased to inform you that your manuscript is accepted for publication and is now being sent to our publisher to be included in the next available issue of EMBO Molecular Medicine!

Please read below for additional IMPORTANT information regarding your article, its publication and the production process.

Congratulations on your interesting work,

With kind regards

Lise

Lise Roth, Ph.D
Senior Editor
EMBO Molecular Medicine

Follow us on Twitter @EmboMolMed
Sign up for eTOCs at embopress.org/alertsfeeds

*** *** *** IMPORTANT INFORMATION *** *** ***

SPEED OF PUBLICATION
The journal aims for rapid publication of papers, using using the advance online publication "Early View" to expedite the process: A properly copy-edited and formatted version will be published as "Early View" after the proofs have been corrected. Please help the Editors and publisher avoid delays by providing e-mail address(es), telephone and fax numbers at which author(s) can be contacted.

Should you be planning a Press Release on your article, please get in contact with embomolmed@wiley.com as early as possible, in order to coordinate publication and release dates.

LICENSE AND PAYMENT:

All articles published in EMBO Molecular Medicine are fully open access: immediately and freely available to read, download and share.

EMBO Molecular Medicine charges an article processing charge (APC) to cover the publication costs. You, as the corresponding author for this manuscript, should have already received a quote with the article processing fee separately. Please let us know in case this quote has not been received.

Once your article is at Wiley for editorial production you will receive an email from Wiley's Author Services system, which will ask you to log in and will present you with the publication license form for completion. Within the same system the publication fee can be paid by credit card, an invoice, pro forma invoice or purchase order can be requested.

Payment of the publication charge and the signed Open Access Agreement form must be received before the article can be published online.

PROOFS

You will receive the proofs by e-mail approximately 2 weeks after all relevant files have been sent o our Production Office. Please return them within 48 hours and if there should be any problems, please contact the production office at embopressproduction@wiley.com.

Please inform us if there is likely to be any difficulty in reaching you at the above address at that time. Failure to meet our

deadlines may result in a delay of publication.

All further communications concerning your paper proofs should quote reference number EMM-2022-17248-V3 and be directed to the production office at embopressproduction@wiley.com.

EMBO Press Author Checklist

Corresponding Author Name: Muriel Le Romancer
Journal Submitted to: Embo Mol Med
Manuscript Number: 17248

USEFUL LINKS FOR COMPLETING THIS FORM
The EMBO Journal - Author Guidelines
EMBO Reports - Author Guidelines
Molecular Systems Biology - Author Guidelines
EMBO Molecular Medicine - Author Guidelines

Reporting Checklist for Life Science Articles (updated January
This checklist is adapted from Materials Design Analysis Reporting (MDAR) Checklist for Authors. MDAR establishes a minimum set of requirements in transparent reporting in the life sciences (see Statement of Task: 10.31222/osf.io/9sm4x). Please follow the journal's guidelines in preparing your
Please note that a copy of this checklist will be published alongside your article.

Abridged guidelines for figures
1. Data
The data shown in figures should satisfy the following conditions:
- the data were obtained and processed according to the field's best practice and are presented to reflect the results of the experiments in an accurate and unbiased manner.
- ideally, figure panels should include only measurements that are directly comparable to each other and obtained with the same assay.
- plots include clearly labeled error bars for independent experiments and sample sizes. Unless justified, error bars should not be shown for technical
- if n<5, the individual data points from each experiment should be plotted. Any statistical test employed should be justified.
- Source Data should be included to report the data underlying figures according to the guidelines set out in the authorship guidelines on Data

2. Captions
Each figure caption should contain the following information, for each panel where they are relevant:
- a specification of the experimental system investigated (eg cell line, species name).
- the assay(s) and method(s) used to carry out the reported observations and measurements.
- an explicit mention of the biological and chemical entity(ies) that are being measured.
- an explicit mention of the biological and chemical entity(ies) that are altered/varied/perturbed in a controlled manner.
- the exact sample size (n) for each experimental group/condition, given as a number, not a range;
- a description of the sample collection allowing the reader to understand whether the samples represent technical or biological replicates (including how many animals, litters, cultures, etc.).
- a statement of how many times the experiment shown was independently replicated in the laboratory.
- definitions of statistical methods and measures:
  - common tests, such as t-test (please specify whether paired vs. unpaired), simple χ^2 tests, Wilcoxon and Mann-Whitney tests, can be unambiguously identified by name only, but more complex techniques should be described in the methods section;
  - are tests one-sided or two-sided?
  - are there adjustments for multiple comparisons?
  - exact statistical test results, e.g., P values = x but not P values < x;
  - definition of 'center values' as median or average;
  - definition of error bars as s.d. or s.e.m.

Please complete ALL of the questions below.
Select "Not Applicable" only when the requested information is not relevant for your study.

Materials

Newly Created Materials	Information included in the manuscript?	In which section is the information available? (Reagents and Tools Table, Materials and Methods, Figures, Data Availability Section)
New materials and reagents need to be available; do any restrictions apply?	Not Applicable	

Antibodies	Information included in the manuscript?	In which section is the information available? (Reagents and Tools Table, Materials and Methods, Figures, Data Availability Section)
For **antibodies** provide the following information:<br>- Commercial antibodies: RRID (if possible) or supplier name, catalogue number and or/clone number<br>- Non-commercial: RRID or citation	Yes	Supp Table 2

DNA and RNA sequences	Information included in the manuscript?	In which section is the information available? (Reagents and Tools Table, Materials and Methods, Figures, Data Availability Section)
**Short novel DNA or RNA including primers, probes**: provide the sequences.	Yes	Supp Table 1

Cell materials	Information included in the manuscript?	In which section is the information available? (Reagents and Tools Table, Materials and Methods, Figures, Data Availability Section)
**Cell lines:** Provide species information, strain. Provide accession number in repository **OR** supplier name, catalog number, clone number, and/**OR** RRID.	Yes	Material and methods cells purchased by ATCC
**Primary cultures:** Provide species, strain, sex of origin, genetic modification status.	Not Applicable	
Report if the cell lines were recently **authenticated** (e.g., by STR profiling) and tested for mycoplasma contamination.	Yes	Yes, we test our cell lines every month

Experimental animals	Information included in the manuscript?	In which section is the information available? (Reagents and Tools Table, Materials and Methods, Figures, Data Availability Section)
**Laboratory animals or Model organisms:** Provide species, strain, sex, age, genetic modification status. Provide accession number in repository **OR** supplier name, catalog number, clone number, **OR** RRID.	Not Applicable	
**Animal observed in or captured from the field:** Provide species, sex, and age where possible.	Not Applicable	
Please detail **housing and husbandry conditions**.	Not Applicable	

Plants and microbes	Information included in the manuscript?	In which section is the information available? (Reagents and Tools Table, Materials and Methods, Figures, Data Availability Section)
**Plants:** provide species and strain, ecotype and cultivar where relevant, unique accession number if available, and source (including location for collected wild specimens).	Not Applicable	
**Microbes:** provide species and strain, unique accession number if available, and source.	Not Applicable	

Human research participants	Information included in the manuscript?	In which section is the information available? (Reagents and Tools Table, Materials and Methods, Figures, Data Availability Section)
If collected and within the bounds of privacy constraints report on age, sex and gender or ethnicity for all study participants.	Yes	Materials and methods

Core facilities	Information included in the manuscript?	In which section is the information available? (Reagents and Tools Table, Materials and Methods, Figures, Data Availability Section)
If your work benefited from core facilities, was their service mentioned in the acknowledgments section?	Yes	Acknowledgements section

Design

Study protocol	Information included in the manuscript?	In which section is the information available? (Reagents and Tools Table, Materials and Methods, Figures, Data Availability Section)
If study protocol has been **pre-registered, provide DOI in the manuscript**. For clinical trials, provide the trial registration number **OR** cite DOI.	Not Applicable	
Report the **clinical trial registration number** (at ClinicalTrials.gov or equivalent), where applicable.	Not Applicable	

Laboratory protocol	Information included in the manuscript?	In which section is the information available? (Reagents and Tools Table, Materials and Methods, Figures, Data Availability Section)
Provide DOI OR other citation details if **external detailed step-by-step protocols** are available.	Not Applicable	

Experimental study design and statistics	Information included in the manuscript?	In which section is the information available? (Reagents and Tools Table, Materials and Methods, Figures, Data Availability Section)
Include a statement about **sample size** estimate even if no statistical methods were used.	Yes	Legend to figures
Were any steps taken to minimize the effects of subjective bias when allocating animals/samples to treatment (e.g. **randomization procedure**)? If yes, have they been described?	Yes	Material and methods
Include a statement about **blinding** even if no blinding was done.	Yes	Material and methods
Describe **inclusion/exclusion criteria** if samples or animals were excluded from the analysis. Were the criteria pre-established?<br><br>If sample or data points were omitted from analysis, report if this was due to attrition or intentional exclusion and provide justification.	Not Applicable	no
For every figure, are **statistical tests** justified as appropriate? Do the data meet the assumptions of the tests (e.g., normal distribution)? Describe any methods used to assess it. Is there an estimate of variation within each group of data? Is the variance similar between the groups that are being statistically compared?	Yes	Legen to figures

Sample definition and in-laboratory replication	Information included in the manuscript?	In which section is the information available? (Reagents and Tools Table, Materials and Methods, Figures, Data Availability Section)
In the figure legends: state number of times the experiment was **replicated** in laboratory.	Yes	Figure legends
In the figure legends: define whether data describe **technical or biological replicates**.	Yes	Figure legends

Ethics

Ethics	Information included in the manuscript?	In which section is the information available? (Reagents and Tools Table, Materials and Methods, Figures, Data Availability Section)
Studies involving **human participants**: State details of **authority granting ethics approval** (IRB or equivalent committee(s), provide reference number for approval.	Yes	Material and Methods
Studies involving **human participants**: Include a statement confirming that **informed consent** was obtained from all subjects and that the experiments conformed to the principles set out in the WMA Declaration of Helsinki and the Department of Health and Human Services Belmont Report.	Yes	Material and Methods
Studies involving **human participants:** For publication of **patient photos**, include a statement confirming that consent to publish was obtained.	Not Applicable	
Studies involving experimental **animals**: State details of **authority granting ethics approval** (IRB or equivalent committee(s), provide reference number for approval. Include a statement of compliance with ethical regulations.	Not Applicable	The experiments on animals were not performed in our lab. We have just collected the parrafin-embedded tumors
Studies involving **specimen and field samples:** State if relevant **permits** obtained, provide details of authority approving study; if none were required, explain why.	Not Applicable	

Dual Use Research of Concern (DURC)	Information included in the manuscript?	In which section is the information available? (Reagents and Tools Table, Materials and Methods, Figures, Data Availability Section)
Could your study fall under dual use research restrictions? Please check biosecurity documents and list of **select agents and toxins** (CDC): https://www.selectagents.gov/sat/list.htm	Not Applicable	
If you used a select agent, is the security level of the lab appropriate and reported in the manuscript?	Not Applicable	
If a study is subject to dual use research of concern regulations, is the name of the **authority granting approval and reference number** for the regulatory approval provided in the manuscript?	Not Applicable	

Reporting

The MDAR framework recommends adoption of discipline-specific guidelines, established and endorsed through community initiatives. Journals have their own policy about requiring specific guidelines and recommendations to complement MDAR.

Adherence to community standards	Information included in the manuscript?	In which section is the information available? (Reagents and Tools Table, Materials and Methods, Figures, Data Availability Section)
State if relevant guidelines or checklists (e.g., **ICMJE, MIBBI, ARRIVE, PRISMA**) have been followed or provided.	Not Applicable	
For **tumor marker prognostic studies,** we recommend that you follow the **REMARK** reporting guidelines (see link list at top right). See author guidelines, under 'Reporting Guidelines'. Please confirm you have followed these guidelines.	Yes	Method section
For **phase II and III randomized controlled trials**, please refer to the **CONSORT** flow diagram (see link list at top right) and submit the CONSORT checklist (see link list at top right) with your submission. See author guidelines, under 'Reporting Guidelines'. Please confirm you have submitted this list.	Not Applicable	

Data Availability

Data availability	Information included in the manuscript?	In which section is the information available? (Reagents and Tools Table, Materials and Methods, Figures, Data Availability Section)
Have **primary datasets** been deposited according to the journal's guidelines (see 'Data Deposition' section) and the respective accession numbers provided in the Data Availability Section?	Not Applicable	
Were **human clinical and genomic datasets** deposited in a public access-controlled repository in accordance to ethical obligations to the patients and to the applicable consent agreement?	Not Applicable	
Are **computational models** that are central and integral to a study available without restrictions in a machine-readable form? Were the relevant accession numbers or links provided?	Not Applicable	
If publicly available data were reused, provide the respective **data citations in the reference list**.	Not Applicable	
